# The Combination of Untargeted Metabolomics with Response Surface Methodology to Optimize the Functional Potential of Common Duckweed (*Lemna minor* L.)

**DOI:** 10.3390/antiox12020313

**Published:** 2023-01-29

**Authors:** Leilei Zhang, Gabriele Rocchetti, Gokhan Zengin, Daniele Del Buono, Marco Trevisan, Luigi Lucini

**Affiliations:** 1 Department for Sustainable Food Process, Università Cattolica del Sacro Cuore, Via Emilia Parmense 84, 29122 Piacenza, Italy; 2 Department of Animal Science, Food, and Nutrition, Università Cattolica del Sacro Cuore, Via Emilia Parmense 84, 29122 Piacenza, Italy; 3 Department of Biology, Faculty of Science, Selcuk University, University Campus, Konya 42130, Turkey; 4 Dipartimento di Scienze Agrarie, Alimentari ed Ambientali, Università Degli Studi di Perugia, Borgo XX Giugno 74, 06121 Perugia, Italy

**Keywords:** untargeted profiling, polyphenols, flavonols, glucosinolates, carotenoids, antioxidant activity, enzyme inhibition activity, nutraceuticals

## Abstract

The present study was designed to evaluate the functional potential of common duckweed (*Lemna minor* L.) as a source of bioactive compounds of nutraceutical interest. The untargeted profiling of the bioactive components of common duckweed was carried out through ultra-high-performance liquid chromatography coupled with high-resolution mass spectrometry (UHPLC-HRMS), in parallel with assessing in vitro antioxidant and enzymatic inhibition properties. The optimization of extraction parameters was determined using the response surface methodology (RSM) through a 3-factor central composite design. The process parameters included extraction temperature, % of ethanol, and ultrasound power, while the response variables were the phenolic content (considering each main phenolic class), total glucosinolates, total carotenoids, the antioxidant potential, and enzyme inhibition activities. The results revealed that common duckweed was a rich source of carotenoids and total flavonoids (mainly flavones and flavonols), followed by phenolic acids, low-molecular-weight phenolics, and glucosinolates. Interestingly, the total flavones, total flavonols and total carotenoid equivalents showed the highest and most positive correlation values with the bioactive properties measured. Finally, the combined RSM approach and unsupervised statistics allowed us to point out the pivotal impact of ethanol percentage in the extraction solvent to recover the highest amounts of bioactive compounds efficiently.

## 1. Introduction

Duckweed (*Lemna minor* L.), also known as water lentil, is a small, free-floating aquatic plant whose fast growth, antimicrobial role, and high nutrient and metal accumulation mean that this species is a potential candidate for phytoremediation research [1]. This plant belongs to the family of Lemnaceae, along with four other aquatic genera (i.e., Spirodela, Landoltia, Wolffia, and Wolffiella), and contains a broad variety of phytochemical constituents, such as amino acids, organic acids, sterols, terpenes, glucosinolates, and phenolic compounds [2,3]. Duckweed has been used for a long time as animal feed, showing no adverse effects. In addition, it is already consumed by humans as a nutritious and sustainable vegetable in Southeast Asian countries, known by the name “Khai-Nam” [4]. According to the literature, *Wolffia arrhiza* and *Wolffia globosa* are mostly consumed in Asian countries, but *L. minor* might have a higher potential as a new sustainable vegetable crop [5]. At the European level, the EFSA Panel on Nutrition, Novel Foods and Food Allergens was asked to deliver an opinion on the safety of water lentil powder (as a dry matrix including 70% of the Lemna genus and 30% of the Wolffia genus) as a novel food pursuant to Regulation (EU) 2015/2283 [6]. The panel stated that based on its chemical composition (mainly when considering its high protein content), the proposed novel food was not nutritionally disadvantageous, except for the concerns regarding the intake of manganese, which represents the main safety concern for the targeted population at very high intakes [6].

On the other hand, as far as the health-promoting benefits of *L. minor* extracts are concerned, limited information is available in the literature regarding the antioxidant and anti-inflammatory potential of *L. minor* lyophilized extracts. For example, it was reported [1] that 45 μg/mL of lyophilized water and ethanol extracts are inhibitors of lipid peroxidation of linoleic acid emulsions, also showing in vitro antioxidant properties, such as radical scavenging and ferrous ion chelating activity. Overall, saponins (23.25 mg/g), flavonoids (0.83 mg/g), and alkaloids (6.40 mg/g) are reported as the main constituents of *L. minor* (on a dry weight basis) [4]. However, as outlined by Xu et al. [4], further studies are needed to gain a better understanding of the health benefits of duckweed phytochemicals. Besides the health implications, there is a growing interest in using new natural additives in the food industry [7,8]. Replacing synthetic additives with natural bioactive compounds extracted from plants is an important strategy for food manufacturers [9]. Additionally, considering that oxidative processes that affect the food matrix are the main non-microbials responsible for quality deterioration [10], it is nowadays pivotal to search for new sustainable sources of phytochemicals with high antioxidant potential and that are easily exploitable in food reformulation.

As a general consideration, it is known that several factors must be considered to obtain a natural extract high in bioactive compounds, such as pH, temperature, the quantity of material, time of extraction and type of solvent [11,12,13]. Therefore, optimizing the maximum number of extraction parameters is extremely important to efficiently recover bioactive compounds. For this purpose, in this work, a multi-response surface methodology (RSM) was used to evaluate the impact of three parameters (temperature, solvent, and ultrasounds’ power) on the comprehensive phenolic (considering the different phenolic classes), carotenoid, and glucosinolate profiles, together with the in vitro antioxidant activity determined through different assays, and enzymatic inhibition of selected enzymes. RSM is a set of mathematical and statistical techniques that describe the functional relationship between one or more responses and a number of independent variables; this technique (developed by Box and Wilson) is now widely used as an optimization tool in many processes, including the extraction of bioactive compounds from plant matrices (based on both conventional and non-conventional methods). Therefore, optimizing the extraction parameters from *L. minor* could be useful to formulate new functional ingredients to be exploited by industry to develop nutraceuticals and foods with improved oxidative stability and health-promoting properties.

## 2. Materials and Methods

### 2.1. Plant Material

Duckweed (*Lemna minor* L.) was collected from a freshwater basin located near the city of Perugia, Italy (43°05′56.1″ N 12°27′29.5″ E). The harvested plants were disinfected by immersion in a 0.5% sodium hypochlorite solution, followed by two rinses in distilled water for 1 min for each step. The plants were then grown in a growth chamber at 23  ±  1 °C, with a light intensity of 100 μmol m^−2^ s^−1^ (light/dark photoperiod: 12/12 h) according to Panfili et al. [14] in polyethylene trays (35 × 28 × 14 cm). The culture media were renewed every two weeks with a 1/10 Hoagland solution. After eight weeks of growth, plant materials were finally collected and lyophilized.

### 2.2. Sample Preparation

In this work, the lyophilized *L. minor* tissues were extracted in ethanol/water at different ratios to isolate more polar compounds. Specifically, 100 mg of dried tissues was extracted in 10 mL of solvent (solid/liquid ratio: 1:100 g/mL) using ultrasound-assisted extraction (UAE; DU-32 ARGOLab, Milan, Italy; maximum power 120 W) at different temperatures and power levels for 20 min. After sonication, the extracts were centrifuged at 6000× *g* for 10 min (4 °C) and then the supernatants were collected and filtered with 0.22 μm syringe filters, while pellets were further extracted with 10 mL of methyl tert-butyl ether (MTBE) to isolate the apolar compound fraction.

### 2.3. Experimental Design and Determination of the Optimum Conditions

The RSM analysis was performed on the software R (version 4.2.1), using the RSM package [15]. The design of the experiment was defined using a central composite design (CCD) [16], considering three independent variables, namely temperature (based on three levels, i.e., 30 °C, 50 °C, and 70 °C), % ethanol (based on three levels: 20%, 50%, and 80%, *v/v*), and ultrasound power based on three different levels, namely 24 (indicated as level 1), 72 (indicated as level 3), and 120 W (indicated as level 5). Accordingly, a total of 21 experimental runs (ERs) was generated. The design points were selected based on considerations of rotability (i.e., the variance in the prediction depends only on the distance from the center) and orthogonality of blocks (i.e., the independency of the block effects on the coefficients of the fitted response surface equation). The experimental data were fitted to the following second-order polynomial model equation:

(1) $$Yj=\beta_{o}+\overset{3}{\underset{i=1}{\sum }}\beta_{i}x_{i}+\sum^{\;}\overset{3}{\underset{i<j=1}{\sum }}\beta_{ij}x_{i}x_{j}+\overset{3}{\underset{i=j}{\sum }}\beta_{ii}x_{i}^{2}$$

where y_j_ is the dependent variable, x_i_ and x_j_ are the independent variables; β_0_, β_i_, β_ij_, and β_ii_ are the regression coefficients. The effects of the temperature (x_1_), the concentration of ethanol (x_2_), and ultrasound power (x_3_) were evaluated in order to optimize the overall recovery of bioactive compounds in the *L. minor* extract, i.e., anthocyanins (y_1_), flavanols (y_2_), other flavonoids (y_3_), flavonols (y_4_), low-molecular-weight (LMW) phenolics (y_5_), phenolic acids (y_6_), stilbenes (y_7_), total flavonoids (y_8_), total phenolics (y_9_), total carotenoids (y_10_), and total glucosinolates (y_11_). Additionally, the RSM model was also used to select the best extraction method that could maximize the antioxidant activity of the extract, thus considering 2,2-difenil-1-picrylidrazyl (DPPH^•^) (y_12_), 2,2′-azino-bis-3-ethylbenzothiazoline-6-sulfonic acid (ABTS^•+^) (y_13_), the cupric-reducing antioxidant capacity (CUPRAC) (y_14_), metal chelating activity (MCA) (y_15_), phosphomolybdenum (PMD) (y_16_), and enzyme inhibitory activity against acetylcholine esterase (AChE) (y_17_), butyrylcholinesterase (BChE) (y_18_), tyrosinase (y_19_), α-amylase (y_20_), and α-glucosidase (y_21_). The adequacy of the model was determined by testing its lack of fit, prediction analysis (such as the canonical analysis), and coefficient of determination (R^2^).

### 2.4. Untargeted Metabolomics Profiling by High-Resolution Mass Spectrometry

#### 2.4.1. Screening of Phenolic Compounds and Glucosinolates

The untargeted phenolic and glucosinolate profiling was performed by high-resolution mass spectrometry on a Q-Exactive™ Focus Hybrid Quadrupole-Orbitrap Mass Spectrometer (Thermo Scientific, Waltham, MA, USA), coupled to a Vanquish ultra-high-pressure liquid chromatography pump and equipped with a heated electrospray ionization (HESI)-II probe (Thermo Scientific, USA). The chromatographic separation consisted of a water–acetonitrile, both liquid chromatography (LC)–mass spectrometry (MS)-grade solutions (from Sigma-Aldrich, Milan, Italy), gradient elution (from 6% up to 94% acetonitrile in 35 min). Additionally, 0.1% formic acid was used as phase modifier. The chromatographic separation was achieved on an Agilent Zorbax Eclipse Plus C18 column (50 × 2.1 mm, 1.8 μm). The HRMS conditions are reported in a previously published work by our research group [17]. The flow rate was 200 μL/min, and the injection volume was 6 μL, using a full-scan data-dependent (top N = 3) MS/MS mode, in the range of 100–1200 *m/z*, with a positive ionization mode and a mass resolution of 70,000 full width half maximum (FWHM). The automatic gain control target (AGC target) and the maximum injection time (IT) of the Orbitrap were 1 × e^6^ and 200 ms, respectively. In the data-dependent MS/MS mode (used for the analysis of the pooled quality control samples), the full-scan mass resolution was reduced to 17,500 FWHM at *m/z* 200, with an AGC target value of 1 × e^5^, maximum IT of 100 ms, and isolation window of 1.0 *m/z*, respectively. The top N ions were fragmented using 10, 20 and 40 eV collisional energies. The HESI parameters are reported in a previous work [17]. The raw data files were further processed using the software MS-DIAL (version 4.70) for post-acquisition data filtering [18], and the annotation was performed via spectral matching against the databases FoodDB and Phenol-Explorer. The identification step was based on mass accuracy (5 ppm tolerance for *m/z* values), isotopic patterns, and spectral matching. These criteria were used to calculate a total identification score, considering the most common source adducts for the chromatographic conditions adopted, thus reaching the level 2 of confidence in annotation [19]. Finally, the cumulative intensity values of the different phenolic and glucosinolate classes annotated were converted into semi-quantitative data, using hydroalcoholic standard solutions of pure compounds (Extrasynthese, Lyon, France) analyzed under the same conditions, namely ferulic acid (phenolic acids), quercetin (flavonols), catechin (flavanols), cyanidin (anthocyanins), luteolin (flavones and other flavonoids), resveratrol (stilbenes), oleuropein (other remaining phenolics), and sulforaphane (total glucosinolates). Linear fitting (R^2^ > 0.98) was used for semi-quantification, expressing the results as μg/g equivalents (Eq.)/g lyophilized extract (n = 3).

#### 2.4.2. Screening of Apolar Compounds

The annotation of apolar compounds (including carotenoids and tocopherols) was performed by UHPLC/HRMS using the same instrument reported in the previous sub-paragraph (Section 2.4.1). The chromatographic separation was achieved using a BEH C18 (2.1 × 100 mm, 1.7 µm) analytical column maintained at 40 °C. The mobile phases consisted of (A) 5 mM ammonium formate and 0.1% formic acid in water/methanol (95/5, *v*/*v*), and (B) 5 mM ammonium formate and 0.1% formic acid in 2-propanol/methanol/water (65/30/5, *v*/*v*/*v*). The linear gradient and flow rate increased linearly as follows, considering the time (min), %B and flow rate (µL/min): (0,10,200), (5, 50, 200), (15, 80, 250), (28, 100, 250), (30, 100, 250), (30.9, 10, 250), and (35, 10, 250). For the full-scan MS analysis, the acquisition was performed using positive and negative ionization with a mass resolution of 70,000 at *m/z* 200. The AGC target and the IT were 1 × e^6^ and 100 ms, respectively. Pooled quality control (QC) samples were prepared and analyzed using a data-dependent (top N = 3) MS/MS mode. The full-scan mass resolution was reduced to 17,500 at *m/z* 200, with an AGC target value of 1 × e^5^, maximum IT of 100 ms, and isolation window of 1.0 *m/z*, respectively. The top N ions were selected for further fragmentation using 10, 20 and 40 eV collisional energies. The injection volume was 6 μL and the *m/z* range for the full-scan analyses was 150–1500. The heated electrospray ionization (HESI) parameters were as follows: sheath gas flow of 30 arb (arbitrary units), auxiliary gas flow of 10 arb, spray voltage of 3.5 kV for ESI + and 2.8 kV for HESI-; capillary temperature of 320 °C. Prior to data collection, the mass spectrometer was calibrated using Pierce™ positive and negative ion calibration solutions (Thermo Fisher Scientific, San Jose, CA, USA). To avoid possible bias, the sequence of injections for Lemna samples was randomized. The collected UHPLC-HRMS data files were then further processed using the software MS-DIAL (version 4.70) [18]. In this regard, automatic peak finding, LOWESS normalization and annotation via spectral matching (against the database FooDB) were performed. The mass range 100–1500 *m/z* was searched for peaks with a minimum peak height of 10,000 cps for ESI+ and ESI− polarities. The MS and MS/MS tolerance for peak centroiding was set to 0.01 and 0.05 Da, respectively. Retention time information was excluded from the calculation of the total score. Accurate mass tolerance for identification was 0.01 Da for MS and 0.05 Da for MS/MS. The identification step was based on mass accuracy, isotopic patterns, and spectral matching. In MS-DIAL, these criteria were used to calculate a total identification score. The total identification score cut-off was 50%, considering the most common ion adducts for a lipidomics workflow. Gap filling using the peak finder algorithm was performed to fill in the missing peaks, considering 5 ppm tolerance for *m/z* values. Finally, to achieve the semi-quantification of carotenoids, the cumulative intensity values were converted into semi-quantitative data using MTBE-standard solutions of pure beta-carotene (Sigma-Aldrich, CAS number: 7235–40-7) analyzed under the same conditions. Linear fitting (R^2^ > 0.98) was used for quantification and the results were expressed as μg equivalents/g dry matter (DM).

### 2.5. In Vitro Assays

#### 2.5.1. Antioxidant Activity

The DPPH^•^ radical scavenging, ABTS^•+^ radical scavenging, cupric ion-reducing antioxidant capacity (CUPRAC), ferric ion-reducing antioxidant power (FRAP), and metal chelating activity were determined, also measuring the total antioxidant activity by phosphomolybdenum method, as accurately reported in a previous work [20]. The in vitro activity data were expressed as µg trolox equivalents (TE)/mL in the DPPH^•^, ABTS^•+^, CUPRAC, and FRAP assay; µg EDTA equivalents (EDTAE)/mL in the metal chelating assay, and mmol TE/mL in the phosphomolybdenum assay.

#### 2.5.2. Enzyme Inhibition Activity

AChE, BChE, tyrosinase, α-amylase, and α-glucosidase inhibition were determined as previously reported by Uysal et al. [20]. The activity data were expressed as µg galantamine (CAS number: 1953-04-4) equivalents (GALAE)/mL in the AChE and BChE assays, µg kojic acid (CAS number: 501-30-4) equivalents (KAE)/mL in the tyrosinase assay, and mmol acarbose (CAS number: 56180-94-0) equivalents (ACAE)/mL in the α-amylase and α-glucosidase assays.

### 2.6. Statistical Analysis

The multivariate data analysis of metabolomics features was performed using two different softwares, namely MetaboAnalyst 5.0 and SIMCA 13 (Umetrics, Malmo, Sweden). Briefly, data were median-centered, Pareto-scaled and log2-transformed before building unsupervised and supervised models, namely hierarchical cluster analysis (HCA; based on the Euclidean distance) and orthogonal projections to latent structures discriminant analysis (OPLS-DA), respectively. The OPLS-DA model was built considering the impact of different % of ethanol, being the most discriminant factor (as highlighted by both HCA and RSM models). The OPLS-DA model validation parameters (namely, goodness-of-fit R^2^ Y and goodness-of-prediction Q^2^ Y) were also recorded. Each discriminant model was inspected for outliers, cross-validated and then permutation testing (N = 200) was performed to prevent over-fitting. The discriminant marker compounds of the different % of ethanol (i.e., 20%, 50%, and 80%) were then evaluated using the variables’ importance in the projection (VIP) selection method, using a VIP score threshold of >1. Pearson’s correlation coefficients (α = 0.05) were then calculated using software R (version 4.2.1).

## 3. Results and Discussion

### 3.1. Phytochemical Profile of L. minor Extracts by UHPLC-HRMS

The untargeted screening of the different duckweed extracts revealed the presence of 367 compounds, including 30 glucosinolates and organosulfur compounds, 38 carotenoids, tocopherols and tocotrienols, and 299 phenolic compounds. Therefore, this analytical approach indicated that the most represented class of compounds in duckweed extracts belonged to polyphenols, with flavonoids showing the highest number of annotated compounds (i.e., 57 flavonols, 52 flavones and other flavonoids, 23 flavanols, and 94 anthocyanins). Additionally, the database Phenol-Explorer allowed us to annotate three other phenolic classes, namely phenolic acids (27 compounds), stilbenes (4 compounds), and other phenolics (42 compounds). In addition, a dedicated tandem MS experiment, by using QC samples and exploiting the comprehensive database FooDB, led us to structurally confirm 42 compounds, including 17 phenolics, 6 glucosinolate derivatives, 4 tocotrienols and tocopherols, and 15 carotenoids (mainly lycopene and carotene derivatives). The detailed list that reports each annotated compound according to a level 2 of confidence, by using both MS and tandem MS acquisitions, together with individual abundance, composite mass spectra and other annotation parameters, is provided in the Supplementary Material (Table S1). In a previous work by Wahman et al. [3], an untargeted analysis (exploiting both TOF and QTOF MS platforms) of *L. minor* metabolites was performed using a workflow based on the utilization of a prioritization tool, coupled with an implemented database for plants (i.e., PLANT-IDENT). Indeed, the authors structurally confirmed the identity of 44 metabolites, mainly amino acids and flavonoids (such as flavonols, flavones, and others). Although overlapping of metabolites could be noticed, exclusive and abundant phenolic compounds could be annotated under our experimental conditions, such as naringenin 6,8-di-*C*-glucoside, isovitexin 2″-(6‴-(E)-*p*-coumaroylglucoside), quercetin 7,4′-*O*-diglucoside, and quercetin 3-*O*-rhamnodiglucoside (Table S1). Regarding the other works available in the scientific literature on *L. minor* metabolites, some key compounds involved in the metabolic response of the plant as survival mechanisms are gallic acid, lignans (such as syringaresinol), flavonoids (such as myricetin and flavone equivalents), and some biflavonoids [21]. Accordingly, our metabolomics workflow outlined the presence of gallic acid and several glycosidic forms of myricetin, followed by some lignans (such as sesaminol, medioresinol, secoisolariciresinol, and matairesinol derivatives). In addition, Kim et al. [22] studied, by using a GC-MS approach, the metabolic profiling of *L. minor* plants cultivated in various concentrations of proline and sucrose, revealing the presence of 46 compounds, including alkaloids, amino acids, fatty acids, organic acids, phenolics, phytosterols, purines and sugars. Therefore, as a general consideration, our analytical approach (based on the analysis of both polar and non-polar fractions and using different combinations of dependent variables) provided us with new insights into the comprehensive composition of *L. minor* extracts, and this was true mainly by looking at the high number of phenolics that were putatively annotated, followed by carotenoids and glucosinolates. Under our experimental conditions, the QC samples showed that the most abundant and structurally confirmed compounds of the duckweed extracts were 10′-apo-beta-caroten-10′-al, quercetin 3-*O*-rhamnodiglucoside, quercetin 7,4′-*O*-diglucoside, methyl 3-(methylthio)butanoate, (2 R)-naringenin 6,8-di-*C*-glucoside, and isovitexin 2″-(6‴-(E)-*p*-coumaroylglucoside). 10′-apo-beta-caroten-10′-al is an apo carotenoid compound that arises from the oxidative degradation of the beta-carotene skeleton at the 10′-position. Specifically, apocarotenoids are metabolites derived from carotenoid enzymatic or non-enzymatic oxidative cleavage. The role of apocarotenoids in gene expression, and modulation of nuclear receptors has been recently reported, suggesting that they are involved in preventing some types of cancer [23]. Apocarotenoids act as precursors of phytohormones, together with being signaling molecules involved in the response to oxidative stress in plants [23]. Methyl 3-(methylthio)butanoate belongs to the class of organosulfur compounds, and specifically, it is a thioester that is likely to be derived from the degradation of methylthio-butyl-glucosinolates [24]. Glucosinolates were previously identified and quantified (recording 4.56 g/kg) in the aqueous extract of *L. minor* by Del Buono et al. [2], showing the abundance of indole derivatives and related breakdown products. A large number of glucosinolate hydrolysis products was found to possess different biological activities (such as anticancer, antioxidant, antifungal, and antimicrobial activities) and the dose dependence of the effects observed makes research in this area both challenging and complex [25]. Regarding the two flavone equivalents that have been structurally confirmed, it was interesting to notice that both belonged to the flavonoid C-glucosides group. According to the literature, C-glycosylation can improve the cellular antioxidation performance of flavonoids (such as apigenin), also eliminating a potential pro-oxidant effect [26]. Isovitexin derivatives are recognized as typical *L. minor* metabolites, and they have been identified in several previous studies that deal with this plant [3,26]. Finally, to the best of our knowledge, this is the first time that quercetin 3-*O*-rhamnodiglucoside and quercetin 7,4′-*O*-diglucoside have been identified in *L. minor* extracts. In this regard, previous phytochemical screening by Wahman et al. [3] revealed only the presence of quercetin and its 3-*O*-glucoside. Taken together, the untargeted screening of the different duckweed extracts revealed a range and abundance of functional compounds such as phenolics, glucosinolates and carotenoids, thus encouraging the next evaluation through RSM methodology regarding the best extraction parameters to obtain a functional extract of nutraceutical interest.

### 3.2. Effect of Extraction Parameters on the Duckweed Extract Properties

The optimization of the extraction conditions from *L. minor* was carried out following an RSM approach. To date, optimizing the extraction method of a plant matrix is extremely important to realize a sustainable and eco-friendly process and reduce the consumption of organic solvents and energy (among others). Therefore, in this study, to maximize the recovery of bioactive compounds, together with the functional properties of the extracts, several factors were considered, including temperature (30, 50, 70 °C), ultrasound power (24, 72, and 120 W, indicated as level 1, 3, and 5, respectively), and concentration of ethanol (20, 50, 80%). Accordingly, Table 1 and Table 2 include the experimental results of each dependent variable (y_n_) obtained by analyzing each of the 21 ERs. As far as the phytochemical profile of the duckweed extracts is concerned (Table 1), we found overall high semi-quantitative contents (expressed as μg/g DM) of phenolics and carotenoids, with phenolic acids (36.6 up to 247.3 μg/g), LMW phenolics (69.9 up to 158.5 μg/g) and flavonols (43.6 up to 158.4 μg/g) being the most abundant compounds detected, also showing a broader concentration range as a function of the different independent variables (x_1_, x_2_, and x_3_). Regarding the different in vitro assays (Table 2), the extracts showed high values of total antioxidant capacity, recording PMD values in the range of 74.0 up to 504.7 mmol TE/g; regarding the enzymatic inhibition values, the duckweed extracts were particularly active against tyrosinase, recording an inhibition activity in the range of 82.4–110.7 mg KAE/g. Lower and comparable inhibition activity values were recorded when considering both α-amylase and α-glucosidase enzymes.

Based on the central composite design results, the regression models were developed to evaluate the associated relationship for the approximation and prediction of responses. Additionally, the statistical significance of the regression coefficients obtained from the analysis of variance (ANOVA) following a second-degree polynomial equation and the determination coefficients (R^2^) was checked and reported in Table 3 and Table 4. The R^2^ values for the different second-order polynomial model equations are reported in Table 3 and Table 4. Overall, by inspecting the R^2^ values, we found that the response of flavanols, flavonols, phenolic acids, stilbenes, carotenoids, and glucosinolates showed high model accuracy, with R^2^ values in the range 0.8352–0.9194, thus suggesting good correlation between the predicted and experimental data. Interestingly, we found a non-significant response for LMW phenolics values (*p* > 0.05; R^2^ = 0.1836; Table 3). Regarding the in vitro assays, we found very low reliability of the developed models for predicting the AChE, tyrosinase, and alpha-glucosidase inhibition activity. On the other hand, extremely significant (*p* < 0.001) and high R^2^ values were recorded when evaluating the behaviour of DPPH^•^ (0.9572), ABTS^•+^ (0.9376), and CUPRAC (0.9564).

Additionally, three-dimensional response surface plots (Figures S1 and S2) and two-dimensional contour plots (Figures S3 and S4) were created, considering the changes in two independent variables to predict the best condition of the extraction process for each of the dependent variables considered. This analysis is very useful to inspect for interaction effects of the factors on the response values, since the three-dimensional response surface plot explains the sensitivity of the response value to the change in the variable. On the other hand, the two-dimensional contour plot describes the significant coefficients among the different variables [27,28]. Overall, the extraction of anthocyanins and flavanols was reported to be highly influenced by ethanol concentration. Particularly, we observed that by employing 40% of ethanol, the highest recovery yield could be obtained, while the ultrasound power factor showed a lower influence (Figures S1A,B and S3A,B). Regarding data on flavonols, LMW phenols and total flavonoids, it has been noted that the extraction efficiency of these compounds was positively influenced by the interaction of temperature and ethanol concentration (Figures S1D,E,H and S3D,E,H). Conversely, stilbene amounts were negatively affected by increasing ethanol ratios (Figures S1G and S3G). Finally, phenolic acids, total phenolics, carotenoids, and glucosinolates were negatively correlated to temperature and ultrasound power, considering that their extraction yield increased as the temperature and ultrasound power parameters decreased (Figures S1F,I–K and S3F,I–K). As far as the in vitro antioxidant activities (i.e., DPPH^•^, ABTS^•+^, and CUPRAC, MCA, and PMD) and enzyme inhibition capacities (against tyrosinase and α-amylase) are concerned, the duckweed extracts obtained by using a high concentration of ethanol and high temperatures showed higher predicted activities (Figures S2A–E,H,I and S4A–E,H,I). On the other hand, the AChE and BChE inhibition capacities were better correlated with hydrophilic duckweed extracts and high temperatures (Figures S2F,G and S4F,G).

The secondary polynomial equations of these models with a higher degree of R^2^ (R^2^ > 0.9) and a significant response (*p* < 0.05) are shown below.

(2) $$Y_{Other\;Flavonoids}=47.14+4.68x_{1}+27.98x_{2}+15.95x_{2}^{2}+7.36x_{3}^{2}+8.27x_{2}x_{3}$$

(3) $$Y_{Glucosinolates}=30.83-1.58x_{3}-14.38x_{2}^{2}+2.57x_{1}x_{2}+2.89x_{2}x_{3}$$

(4) $$Y_{DPPH•}=15.99+1.10x_{1}+5.76x_{2}+0.59x^{2}+5.31x_{2}^{2}-1.18x_{1}x_{2}$$

(5) $$Y_{ABTS•+}=7.19+2.44x_{1}+9.77x_{2}+4.45x_{2}^{2}$$

(6) $$Y_{CUPRAC}=21.83+1.52x_{1}+9.35x_{2}+2.65x_{2}^{2}$$

**Figure 1:**
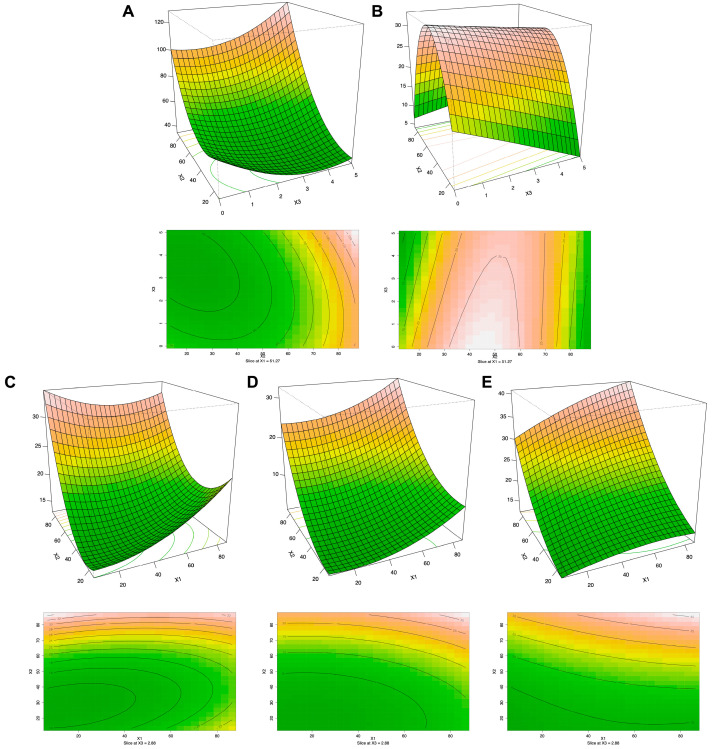
Three-dimensional response surface plots and two-dimensional contour plots that consider the (**A**) other flavonoid and (**B**) glucosinolate phytochemical profile, and (**C**) DPPH^•^, (**D**) ABTS^•+^, and (**E**) CUPRAC antioxidant activities of the 21 duckweed extracts, as a function of extraction temperature, ultrasound power, and % of ethanol.

The analysis of the secondary polynomial equation for the class of “other flavonoids” (2) showed that their extraction yield was influenced positively by temperature (x_1_), % of ethanol (x_2_), and the interaction between % ethanol and ultrasound power (x_2_ x_3_). Indeed, as reported in Table 1, it can be observed that different ethanol concentrations had important effects on the yield of other flavonoids. In addition, the highest extraction yield was detected when a higher concentration of ethanol was used as the extraction solvent, which was reported in the case of the ER4 (116.4 μg/g) and ER17 (116.5 μg/g), employing higher values of temperature and % of ethanol, followed by ER6 (119.3 μg/g) and ER18 (117.5 μg/g), obtained from the interaction between higher values of ethanol ultrasound power. The RSM model predicted an increased extraction yield as the x_2_ and x_3_ parameters increased, reaching a concentration greater than 120 μg/g, as shown in the three-dimensional response surface plots and two-dimensional contour plots (Figure 1A). Accordingly, the hydroalcoholic solution, as well as temperature and ultrasound-assisted extraction techniques, were previously studied and considered to enhance the recovery of phenolic compounds from agri-food waste [29]. The extraction of glucosinolates (3) was negatively affected by ultrasound power and % of ethanol. In addition, a positive interaction was observed between temperature and % of ethanol (x_1_ x_2_) or % of ethanol and ultrasound power (x_2_ x_3_). Indeed, the ERs that had a high recovery rate of glucosinolates were ER1 (30.8 μg/g), ER9 (30.3 μg/g), ER12 (30.5 μg/g), ER16 (30.0 μg/g), and ER20 (31.3 μg/g), obtained from the intermediate level of both temperature and concentration of ethanol. Instead, ER7 (32.0 μg/g) reported a high recovery rate of glucosinolates, obtained from the interaction between the intermediate ethanol concentration and high ultrasound power.

Interestingly, the RSM predicted that the highest extraction yield could be achieved at a medium ethanol concentration in combination with low ultrasound power (Figure 1B). This phenomenon could be explained by the fact that solvent viscosity decreases by increasing the temperature, thus promoting the release of bioactive compounds from the plant matrix. However, it is important to state that high temperature values could also promote the degradation of some temperature-sensitive compounds. In this regard, Doheny-Adams et al. [30] studied three methods of glucosinolates extraction from brassicaceous tissues, using cold methanol, boiling methanol, and boiling water. They highlighted that the extraction method based on higher temperatures was worse than the methods based on the cold temperatures, and lyophilization treatment led to a reduction in the final glucosinolates yield. This observation was also confirmed by other works available in the scientific literature [31,32,33]. Additionally, the in vitro antioxidant activities expressed as DPPH^•^ (4), ABTS^•+^ (5), and CUPRAC (6) were affected by temperature and % of ethanol (Figure 1C–E). Indeed, the ERs that reported the highest in vitro antioxidant activities were ER3, ER4, ER6, ER13, ER17 and ER18, all sharing the same high ethanol concentration (80%).

### 3.3. Multivariate Statistics and Discriminant Marker Compounds

**Figure 2:**
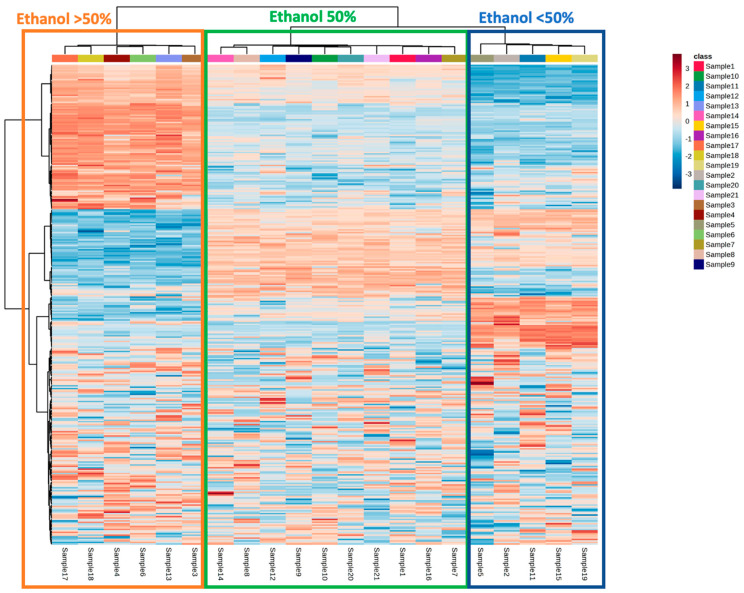
Unsupervised hierarchical cluster analysis (HCA) carried out considering the phytochemical profile of the different 21 duckweed extracts as a function of extraction temperature, ultrasound power, and % of ethanol.

In order to hierarchically assess the impact of the three independent variables on the untargeted phytochemical profile of *L. minor* extracts, we carried out an unsupervised hierarchical statistical analysis, thus inspecting the corresponding heat map (Figure 2).

**Figure 3:**
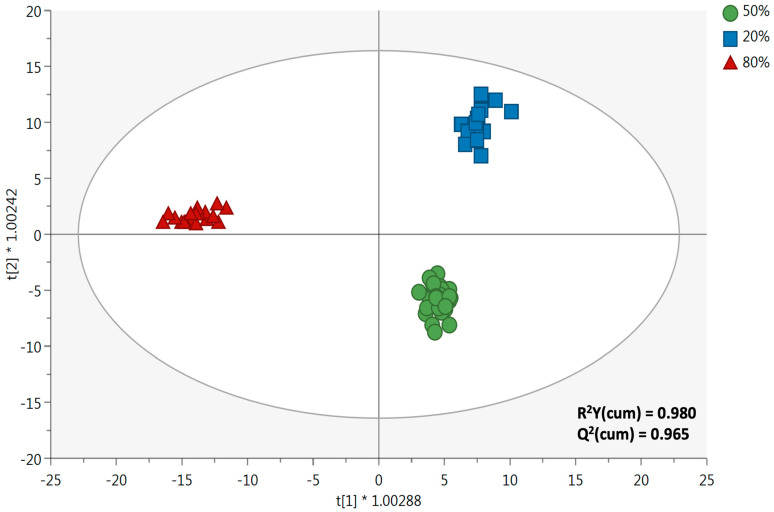
Supervised orthogonal projections to latent structures discriminant analysis (OPLS-DA) score plot, which shows the different duckweed extracts as a function of the % of ethanol used (i.e., 20%, 50%, and 80%). t[1] * 1.00288 and t[2] * 1.00242 are the latent vectors of the OPLS-DA models, used to build the OPLS-DA score plot.

Interestingly, the heat map revealed that the % of ethanol had a hierarchically higher impact in driving the modifications of the phytochemical profile of the duckweed extracts, compared to extraction temperature and ultrasound power. In this regard, two clusters and three subclusters could be identified, corresponding to ethanol 80% (orange), ethanol 50% (green), and ethanol 20% (blue) (Figure 2). Additionally, looking at the heat map, the different % of ethanol used were found to promote the extraction of a specific cluster of compounds, and this was true mainly when considering the 80% and 20% subclusters. Therefore, as the next step, the impact of different % of ethanol was evaluated through a supervised multivariate statistical approach, namely orthogonal projection to latent structures discriminant analysis (OPLS-DA), followed by the selection of the most discriminant variables (VIP approach), as reported in Figure 3. The OPLS-DA score plot showed a clear separation along the orthogonal latent vector as a function of a higher % of ethanol, thus confirming the outcome of the HCA. Furthermore, the supervised model was characterized by extremely significant goodness-of-fitting and goodness-of-prediction values, as R^2^ Y (cum) = 0.980 and the Q^2^ (cum) = 0.965, respectively. In addition, the prediction model was excluded for both outliers (according to Hotelling’s T^2^ test) and overfitting (according to permutation testing; N = 200 random permutations) (Table S1).

The VIP selection method, following the OPLS-DA modelling, allowed us to list 165 potential discriminant metabolites that belong to carotenoids (22 compounds), glucosinolates (14 compounds), polyphenols (123 compounds), tocotrienols (4 compounds), and tocopherols (2 compounds). These metabolites showed a VIP score (a measure of the discrimination potential) of >1, representing those compounds most affected by the different % of ethanol used. To simplify the data elaboration, we reported in Table 5 the most discriminant compounds (i.e., those with the highest VIP score) when considering each class separately.

Overall, it was interesting to notice that three out of four of the most discriminant metabolites were exclusive markers of 50% ethanol, namely methyl 3-(methylthio)butanoate, 7-hydroxysecoisolariciresinol (a lignan), and 1′-carboxy-gamma-tocotrienol. The fourth most discriminant metabolite was the apocarotenoid 2′-apo-beta-carotenal, a specific marker of the 80% ethanol cluster. Furthermore, a detailed overview of the VIP marker compounds (Table S1) outlined that 40.6% belonged to the 80% ethanol group, followed by 30.3% and 29.1% for duckweed samples extracted with 50% and 20% ethanol, respectively. In addition, as expected, water-soluble phenolics (such as anthocyanins, recording 14 compounds) were markers of the 20% ethanol group, while flavones and flavonols were specific markers of the 80% ethanol group. Regarding glucosinolates, the 14 VP compounds detected were particularly sensitive to different percentages of ethanol (Table S1); for example, glucosinalbin 4-rhamnoside (VIP score = 1.60) was a marker of the 20% ethanol group, while glucochlearin (VIP score = 1.44) was a marker of the 80% ethanol group. Therefore, the multivariate statistics provided evidence for the utilization of percentages of ethanol higher than 50% to recover several bioactive compounds that could be exploited by the food science and technology area and are characterized by a potential nutraceutical interest.

### 3.4. Pearson’s Correlations and Canonical Analysis

**Figure 4:**
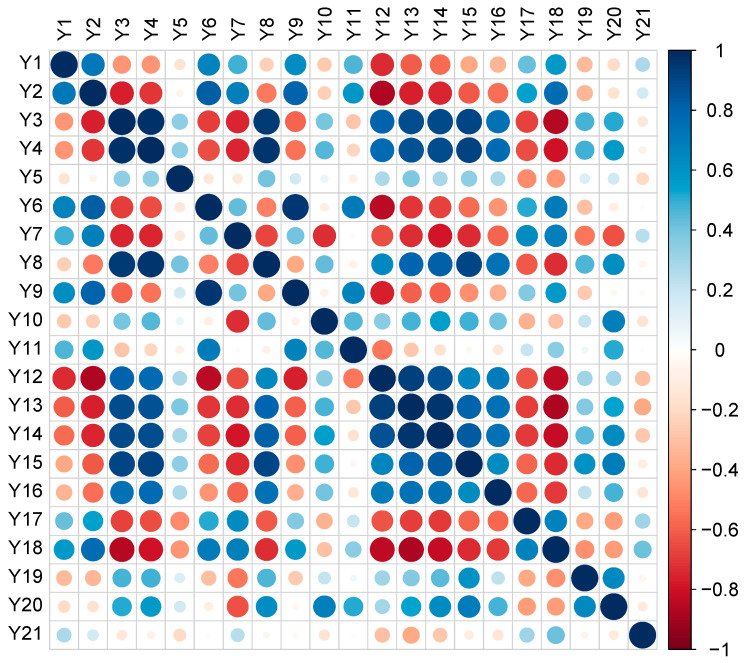
Pearson’s correlation coefficients-based matrix that considers the different dependent variables measured. Blue dots: positive correlations; red dots: negative correlations. Anthocyanins (y_1_), flavanols (y_2_), other flavonoids (y_3_), flavonols (y_4_), low-molecular-weight (LMW) phenolics (y_5_), phenolic acids (y_6_), stilbenes (y_7_), total flavonoids (y_8_), total phenolics (y_9_), total carotenoids (y_10_), total glucosinolates (y_11_), DPPH^•^ (y_12_), ABTS^•+^ (y_13_), CUPRAC (y_14_), MCA (y_15_), PMD (y_16_), AChE (y_17_), BChE (y_18_), tyrosinase (y_19_), α-amylase (y_20_), and α-glucosidase (y_21_).

Pearson’s correlations between the total phenolic content, total carotenoids, total glucosinolates and antioxidant activities and enzymatic inhibition capacities were evaluated and graphically and numerically presented in Figure 4 and Table S2, respectively. Overall, high correlation coefficients among other flavonoids (y3) and flavonols (y4), as well as total flavonoids (y8) and radical scavenging activities, i.e., DPPH^•^ (y_12_), ABTS^•+^ (y_13_), CUPRAC (y_14_), MCA (y_15_) and PMD (y_16_), have been recorded (r > 0.7, *p* < 0.05). In contrast, negative correlations were observed against AChE (y_17_) and BChE (y_18_) (r > −0.6, *p* < 0.05). On the contrary, anthocyanins (y_1_), flavanols (y_2_), phenolic acids (y_6_), and stilbenes (y_7_) were negatively correlated with in vitro antioxidant activities, outlining strong negative correlations against DPPH^•^ (y_12_) and ABTS^•+^ (y_13_) (Figure 4 and Table S2). Interestingly, no significant correlation coefficients were recorded when considering the tyrosinase (y_19_), α-amylase (y_20_), and α-glucosidase (y_21_) inhibition capacity values against the classes of compounds considered.

Finally, with the final aim of improving the recovery of bioactive compounds, as well as the biological activities, a canonical analysis was used to record the optimum extraction conditions. Indeed, the optimal values of the selected variables were obtained by solving the regression Equation (2) for each dependent variable, where the reliability of the predicted condition was in accordance with the R^2^ of the model (Table 3 and Table 4). In addition, the optimum conditions for each independent variable considered are reported in Table 6. According to the detected Pearson’s correlation coefficients, the group of “other flavonoids” and “flavonols” were strictly correlated to the duckweed extract’s in vitro antioxidant capacity. The RSM model successfully predicted the best extraction conditions to maximize the radical scavenger capacities. The predicted conditions to reach the highest concentrations of other flavonoids (i.e., 154.68 μg/g) were as follows: extraction temperature equal to 58.98 °C, ethanol concentration equal to 97.95%, and ultrasound power equal to 98.88 W. Similar predicted conditions were obtained for flavonols (reaching 190.25 μg/g), including an extraction temperature equal to 60.30 °C, ethanol concentration equal to 97.52%, and ultrasound power equal to 101.04 W. Therefore, the optimal operational conditions could be modified as follows: extraction temperature: 50–60 °C, ethanol concentration: 95–97%, and ultrasound power: 96 W.

## 4. Conclusions

In addition to its potential as a sustainable nutritional ingredient, duckweed has been characterized as a promising source of functional components. Nowadays, the combination of a favorable profile in terms of sustainability, with health-promoting bioactive components, is particularly interesting for the food industry. This latter trait has great potential in terms of further exploitation as a nutraceutical or as a food ingredient and food additive. However, standardizing and optimizing the yields of functional compounds is a primary goal to ensure effective implementation procedures. In this framework, the very comprehensive profiling ensured by untargeted metabolomics (which focused on both polar and apolar compounds) coupled with the response surface methodology data management has provided, for the first time, the optimal conditions to recover functional components from duckweed. Although the effect of plant growth conditions (including light, plant nutrition and edaphic factors) still remains as key information that has not been properly addressed yet, our results provide novel information about the phytochemical diversity of duckweed and its corresponding functional potential, thus supporting the further exploitation of this promising source of bioactives for food-related applications (e.g., as an extender of meat and meat products).

## Figures and Tables

**Table 1:** Central composite design and experimental results obtained for dependent variables *.

	T (°C)	EtOH (%)	Power Level	Anthocyanins	Flavanols	Other Flavonoids	Flavonols	LMW Phenolics	Phenolic Acids	Stilbenes	Total Flavonoids	Total Phenolics	Carotenoids	Glucosinolates
	Independent Variables			Dependent Variables										
	*x_1_*	*x_2_*	*x_3_*	*y_1_*	*y_2_*	*y_3_*	*y_4_*	*y_5_*	*y_6_*	*y_7_*	*y_8_*	*y_9_*	*y_10_*	*y_11_*
1	50 (0)	50 (0)	3 (0)	9.4 ± 1.0 ^abc^	32.7 ± 3.5 ^abcd^	46.9 ± 3.8 ^c^	64.4 ± 15.9 ^de^	104.5 ± 14.8	226.1 ± 48.4 ^ab^	2.8 ± 0.2 ^cdefg^	153.5 ± 24.1 ^ef^	333.3 ± 63.0 ^ab^	229.6 ± 23.8 ^de^	30.8 ± 3.3 ^a^
2	30 (−1)	20 (−1)	3 (0)	8.3 ± 2.6 ^abc^	36.9 ± 11.4 ^ab^	40.4 ± 3.4 ^c^	47.8 ± 31.3 ^e^	108.9 ± 48.5	168.8 ± 79.1 ^abcde^	3.9 ± 0.2 ^abc^	133.4 ± 45.4 ^ef^	281.6 ± 106.5 ^ab^	21.5 ± 3.0 ^h^	13.5 ± 1.5 ^c^
3	50 (0)	80 (1)	1 (−1)	6.0 ± 2.7 ^bc^	3.6 ± 0.7 ^e^	90.5 ± 8.8 ^ab^	125.3 ± 23.3 ^abcd^	98.7 ± 18.2	62.9 ± 29.6 ^de^	2.0 ± 0.0 ^g^	225.5 ± 33.4 ^abcde^	163.6 ± 16.6 ^b^	251.9 ± 8.8 ^cd^	14.9 ± 6.4 ^c^
4	70 (1)	80 (1)	3 (0)	8.0 ± 1.8 ^abc^	3.6 ± 0.8 ^e^	116.4 ± 16.0 ^a^	150.9 ± 41.6 ^a^	110.3 ± 5.8	84.8 ± 61.0 ^bcde^	2.0 ± 0.0 ^g^	279.0 ± 57.7 ^abc^	197.1 ± 59.3 ^ab^	188.2 ± 7.4 ^g^	18.1 ± 10.1 ^bc^
5	50 (0)	20 (−1)	5 (1)	11.5 ± 3.5 ^ab^	31.1 ± 1.4 ^abcde^	36.8 ± 1.8 ^c^	43.6 ± 28.4 ^e^	97.9 ± 44.7	177.5 ± 6.1 ^abcde^	3.3 ± 0.3 ^bcde^	123.0 ± 28.6 ^f^	278.7 ± 39.9 ^ab^	20.1 ± 3.6 ^h^	12.6 ± 1.1 ^c^
6	50 (0)	80 (1)	5 (1)	8.9 ± 0.9 ^abc^	6.3 ± 0.9 ^cde^	119.3 ± 10.8 ^a^	148.4 ± 47.9 ^ab^	158.5 ± 26.8	64.1 ± 23.0 ^de^	2.0 ± 0.0 ^g^	283.1 ± 59.0 ^ab^	224.7 ± 36.1 ^ab^	196.2 ± 7.4 ^g^	14.9 ± 5.4 ^c^
7	30 (−1)	50 (0)	5 (1)	9.8 ± 2.8 ^abc^	31.7 ± 7.8 ^abcde^	50.6 ± 3.4 ^c^	69.0 ± 7.1 ^cde^	93.0 ± 21.1	211.6 ± 9.2 ^abc^	3.0 ± 0.2 ^bcdefg^	161.2 ± 18.4 ^ef^	307.6 ± 23.8 ^ab^	247.7 ± 10.8 ^cd^	32.0 ± 1.8 ^a^
8	70 (1)	50 (0)	5 (1)	9.9 ± 1.2 ^abc^	44.4 ± 13.7 ^ab^	53.9 ± 1.8 ^c^	72.4 ± 2.9 ^cde^	104.1 ± 10.4	141.5 ± 60.8 ^abcde^	3.1 ± 0.0 ^bcdefg^	180.8 ± 16.9 ^cdef^	248.6 ± 51.6 ^ab^	243.7 ± 6.7 ^cd^	29.4 ± 1.3 ^ab^
9	50 (0)	50 (0)	3 (0)	10.1 ± 0.9 ^abc^	50.2 ± 8.2 ^ab^	50.9 ± 4.0 ^c^	72.5 ± 8.9 ^cde^	145.4 ± 35.9	213.3 ± 11.9 ^abc^	2.6 ± 0.1 ^defg^	183.8 ± 11.5 ^bcdef^	361.3 ± 47.8 ^a^	200.2 ± 5.8 ^fg^	30.3 ± 0.8 ^a^
10	50 (0)	20 (−1)	1 (−1)	9.7 ± 0.9 ^abc^	48.3 ± 6.4 ^ab^	41.9 ± 12.7 ^c^	73.5 ± 11.4 ^cde^	95.0 ± 11.5	167.2 ± 16.7 ^abcde^	3.4 ± 0.3 ^bcde^	173.3 ± 8.3 ^def^	265.6 ± 26.3 ^ab^	208.9 ± 9.1 ^efg^	27.1 ± 4.4 ^ab^
11	70 (1)	20 (−1)	3 (0)	10.8 ± 0.8 ^ab^	40.4 ± 3.5 ^ab^	38.2 ± 5.2 ^c^	54.4 ± 9.5 ^de^	125.6 ± 92.1	170.8 ± 15.0 ^abcde^	4.2 ± 1.1 ^ab^	143.8 ± 10.4 ^ef^	300.7 ± 96.3 ^ab^	19.9 ± 1.5 ^h^	9.3 ± 4.3 ^c^
12	30 (−1)	50 (0)	1 (−1)	11.4 ± 2.8 ^ab^	36.3 ± 10.7 ^ab^	49.9 ± 2.4 ^c^	66.7 ± 15.4 ^de^	108.1 ± 38.9	247.3 ± 11.5 ^a^	2.4 ± 0.1 ^efg^	164.3 ± 11.9 ^ef^	357.8 ± 147.3 ^a^	394.5 ± 12.4 ^a^	30.5 ± 0.9 ^a^
13	30 (−1)	80 (1)	3 (0)	4.3 ± 1.6 ^c^	3.2 ± 1.5 ^e^	64.5 ± 14.3 ^bc^	93.2 ± 29.6 ^abcde^	122.7 ± 19.5	36.6 ± 5.9 ^e^	2.0 ± 0.0 ^fg^	165.2 ± 19.4 ^ef^	161.4 ± 17.5 ^b^	386.8 ± 12.5 ^a^	14.9 ± 5.2 ^c^
14	70 (1)	50 (0)	1 (−1)	10.9 ± 2.6 ^ab^	47.7 ± 12.9 ^ab^	53.2 ± 5.5 ^c^	77.8 ± 9.5 ^bcde^	98.9 ± 36.5	171.2 ± 42.4 ^abcde^	3.5 ± 0.8 ^abcde^	189.6 ± 26.3 ^abcdef^	273.7 ± 14.5 ^ab^	189.9 ± 3.9 ^g^	28.6 ± 0.3 ^ab^
15	50 (0)	50 (0)	3 (0)	10.2 ± 2.3 ^abc^	27.4 ± 8.5 ^bcde^	39.6 ± 4.1 ^c^	50.2 ± 12.4 ^e^	69.9 ± 8.7	131.2 ± 57.3 ^abcde^	3.3 ± 0.2 ^bcdef^	127.5 ± 24.4 ^ef^	204.4 ± 52.4 ^ab^	22.3 ± 2.2 ^h^	12.0 ± 2.3 ^c^
16	50 (0)	50 (0)	3 (0)	11.5 ± 0.7 ^ab^	28.1 ± 8.5 ^bcde^	52.1 ± 1.9 ^c^	66.1 ± 23.2 ^de^	91.4 ± 21.3	152.9 ± 58.3 ^abcde^	3.1 ± 0.6 ^bcdefg^	157.8 ± 18.6 ^ef^	247.4 ± 77.0 ^ab^	211.9 ± 5.6 ^efg^	30.0 ± 2.0 ^a^
17	70 (1)	80 (1)	1 (−1)	8.2 ± 1.7 ^abc^	3.6 ± 1.1 ^e^	116.5 ± 20.4 ^a^	158.4 ± 12.8 ^a^	127.7 ± 19.3	70.9 ± 36.9 ^cde^	2.0 ± 0.0 ^g^	286.7 ± 32.3 ^a^	200.6 ± 53.1 ^ab^	290.3 ± 9.5 ^b^	13.3 ± 1.4 ^c^
18	30 (−1)	80 (1)	5 (1)	7.8 ± 2.5 ^abc^	5.7 ± 0.9 ^de^	117.5 ± 18.9 ^a^	139.9 ± 28.4 ^abc^	124.5 ± 12.8	42.9 ± 7.4 ^e^	2.1 ± 0.0 ^fg^	270.9 ± 45.5 ^abcd^	169.4 ± 5.5 ^b^	269.6 ± 1.9 ^bc^	12.0 ± 0.8 ^c^
19	70 (1)	20 (−1)	1 (−1)	10.0 ± 2.3 ^abc^	35.1 ± 3.3 ^abc^	38.0 ± 6.9 ^c^	50.3 ± 17.2 ^e^	116.0 ± 63.3	120.9 ± 70.1 ^abcde^	4.7 ± 0.5 ^a^	133.5 ± 24.9 ^ef^	241.7 ± 43.3 ^ab^	20.1 ± 1.1 ^h^	13.1 ± 0.5 ^c^
20	50 (0)	50 (0)	3 (0)	9.7 ± 2.1 ^abc^	45.2 ± 28.2 ^ab^	52.9 ± 9.0 ^c^	70.6 ± 5.1 ^cde^	140.4 ± 49.2	182.3 ± 31.1 ^abcde^	3.3 ± 0.6 ^bcde^	178.3 ± 36.7 ^def^	326.1 ± 34.3 ^ab^	266.9 ± 4.3 ^bc^	31.3 ± 3.9 ^a^
21	50 (0)	50 (0)	5 (1)	12.8 ± 0.8 ^a^	57.7 ± 8.0 ^a^	58.2 ± 11.1 ^c^	78.7 ± 30.2 ^bcde^	110.5 ± 48.34	207.1 ± 16.7 ^abcd^	3.8 ± 0.2 ^abcd^	207.4 ± 49.4 ^abcdef^	321.4 ± 45.3 ^ab^	225.4 ± 3.6 ^def^	28.9 ± 0.8 ^ab^

* Results are expressed as mean value (as μg/g equivalents (Eq.)/g lyophilized extract) ± standard deviation (n = 3) and reported as dry weight (DM). Different letters in the same column indicate significant differences resulting from ANOVA; *p* < 0.05—Tukey’s HSD post hoc test.

**Table 2:** Central composite design and experimental results obtained for dependent variables *.

	T (°C)	EtOH (%)	Power Level	DPPH^•^	ABTS^•+^	CUPRAC	MCA	PMD	AChE	BChE	Tyrosinase	α-Amylase	α-Glucosidase
	Independent Variables			Dependent Variables									
	*x* *_1_*	*x* *_2_*	*x* *_3_*	*y* *_12_*	*y* *_13_*	*y* *_14_*	*y* *_15_*	*y* *_16_*	*y* *_17_*	*y* *_18_*	*y* *_19_*	*Y* *_20_*	*y* *_21_*
1	50 (0)	50 (0)	3 (0)	16.3 ± 0.6 ^ef^	6.6 ± 0.7 ^d^	21.7 ± 0.2 ^ef^	29.6 ± 2.7 ^def^	193.7 ± 0.6 ^cd^	5.7 ± 0.02 ^ab^	7.3 ± 0.01 ^ef^	97.3 ± 0.8 ^ef^	0.42 ± 0.00 ^bcde^	1.94 ± 0.00 ^abc^
2	30 (−1)	20 (−1)	3 (0)	14.8 ± 0.6 ^f^	1.6 ± 0.4 ^f^	15.3 ± 0.4 ^i^	28.0 ± 0.5 ^fg^	117.9 ± 7.7 ^fghi^	5.6 ± 0.01 ^bcde^	7.7 ± 0.06 ^cd^	94.3 ± 2.3 ^fgh^	0.32 ± 0.01 ^g^	1.94 ± 0.00 ^abc^
3	50 (0)	80 (1)	1 (−1)	27.4 ± 0.8 ^bc^	21.9 ± 0.8 ^b^	33.3 ± 0.6 ^c^	39.5 ± 0.5 ^a^	216.8 ± 5.9 ^bc^	5.6 ± 0.03 ^bcde^	7.2 ± 0.01 ^f^	107.6 ± 0.7 ^abc^	0.43 ± 0.01 ^ab^	1.94 ± 0.00 ^abc^
4	70 (1)	80 (1)	3 (0)	26.0 ± 0.9 ^c^	22.3 ± 0.7 ^b^	34.8 ± 1.0 ^b^	40.8 ± 0.4 ^a^	208.5 ± 1.2 ^bc^	5.6 ± 0.02 ^bcde^	6.6 ± 0.11 ^g^	107.7 ± 0.8 ^ab^	0.42 ± 0.01 ^ab^	1.94 ± 0.01 ^abc^
5	50 (0)	20 (−1)	5 (1)	15.1 ± 0.9 ^f^	0.4 ± 0.0 ^f^	14.8 ± 0.1 ^i^	28.1 ± 0.7 ^fg^	74.0 ± 1.8 ^l^	5.7 ± 0.01 ^ab^	8.1 ± 0.0 ^a^	100.6 ± 1.2 ^de^	0.27 ± 0.01 ^h^	1.95 ± 0.00 ^ab^
6	50 (0)	80 (1)	5 (1)	24.9 ± 1.0 ^c^	23.8 ± 0.8 ^ab^	34.6 ± 0.4 ^bc^	40.8 ± 0.5 ^a^	230.1 ± 11.2 ^b^	5.4 ± 0.02 ^f^	6.4 ± 0.09 ^h^	107.1 ± 0.2 ^abc^	0.42 ± 0.02 ^abc^	1.91 ± 0.01 ^d^
7	30 (−1)	50 (0)	5 (1)	15.4 ± 0.3 ^ef^	3.7 ± 0.1 ^e^	17.5 ± 0.4 ^h^	34.0 ± 0.3 ^b^	117.3 ± 7.1 ^fghi^	5.7 ± 0.03 ^abcd^	8.2 ± 0.11 ^a^	102.9 ± 0.4 ^cd^	0.39 ± 0.01 ^cdef^	1.95 ± 0.00 ^abc^
8	70 (1)	50 (0)	5 (1)	16.4 ± 1.2 ^ef^	8.2 ± 0.7 ^cd^	22.4 ± 0.2 ^e^	34.4 ± 0.4 ^b^	147.5 ± 2.9 ^e^	5.7 ± 0.04 ^abcd^	7.5 ± 0.07 ^de^	103.1 ± 1.8 ^bcd^	0.42 ± 0.01 ^abcd^	1.92 ± 0.01 ^cd^
9	50 (0)	50 (0)	3 (0)	15.6 ± 0.3 ^ef^	7.3 ± 0.2 ^d^	20.4 ± 0.4 ^fg^	32.4 ± 0.1 ^bcd^	132.1 ± 5.3 ^efgh^	5.6 ± 0.04 ^cde^	7.6 ± 0.11 ^cd^	110.7 ± 0.5 ^a^	0.43 ± 0.01 ^ab^	1.95 ± 0.00 ^ab^
10	50 (0)	20 (−1)	1 (−1)	16.4 ± 1.5 ^ef^	6.9 ± 0.8 ^d^	19.9 ± 0.1 ^g^	32.0 ± 0.2 ^cde^	129.7 ± 5.3 ^efgh^	5.6 ± 0.05 ^abcd^	8.1 ± 0.04 ^a^	103.8 ± 1.3 ^bcd^	0.45 ± 0.02 ^a^	1.96 ± 0.00 ^a^
11	70 (1)	20 (−1)	3 (0)	19.9 ± 2.2 ^d^	6.6 ± 0.7 ^d^	14.9 ± 0.2 ^i^	25.8 ± 0.8 ^g^	112.4 ± 10.8 ^ghi^	5.6 ± 0.05 ^abcd^	8.0 ± 0.11 ^abc^	82.4 ± 0.2 ^m^	0.26 ± 0.02 ^h^	1.93 ± 0.02 ^bcd^
12	30 (−1)	50 (0)	1 (−1)	14.8 ± 0.6 ^f^	7.4 ± 0.1 ^d^	19.9 ± 0.4 ^g^	32.2 ± 2.6 ^bcde^	133.9 ± 11.8 ^efg^	5.7 ± 0.04 ^abc^	8.2 ± 0.09 ^a^	84.8 ± 1.6 ^lm^	0.37 ± 0.00 ^f^	1.95 ± 0.02 ^abc^
13	30 (−1)	80 (1)	3 (0)	29.7 ± 0.3 ^ab^	22.0 ± 0.7 ^b^	33.8 ± 0.2 ^bc^	33.2 ± 0.7 ^bc^	181.3 ± 5.9 ^d^	5.5 ± 0.07 ^de^	7.1 ± 0.06 ^f^	94.6 ± 1.1 ^fgh^	0.38 ± 0.00 ^f^	1.94 ± 0.00 ^abc^
14	70 (1)	50 (0)	1 (−1)	16.7 ± 1.0 ^def^	9.4 ± 0.6 ^c^	25.0 ± 0.4 ^d^	30.4 ± 2.1 ^cdef^	177.7 ± 8.3 ^d^	5.7 ± 0.03 ^abc^	8.3 ± 0.08 ^a^	89.2 ± 2.4 ^il^	0.39 ± 0.00 ^def^	1.95 ± 0.00 ^ab^
15	50 (0)	50 (0)	3 (0)	15.8 ± 0.9 ^ef^	0.9 ± 0.0 ^f^	14.9 ± 0.2 ^i^	27.9 ± 0.1 ^fg^	106.6 ± 17.8 ^hi^	5.6 ± 0.03 ^bcde^	8.0 ± 0.08 ^ab^	87.9 ± 0.4 ^il^	0.27 ± 0.00 ^h^	1.95 ± 0.00 ^ab^
16	50 (0)	50 (0)	3 (0)	15.8 ± 1.1 ^ef^	7.9 ± 0.7 ^cd^	22.3 ± 0.1 ^e^	32.3 ± 0.9 ^bcd^	141.0 ± 7.1 ^ef^	5.6 ± 0.04 ^abcd^	7.8 ± 0.08 ^cd^	90.7 ± 2.5 ^hi^	0.38 ± 0.01 ^f^	1.96 ± 0.00 ^a^
17	70 (1)	80 (1)	1 (−1)	30.9 ± 1.3 ^a^	25.5 ± 1.2 ^a^	36.5 ± 0.4 ^a^	39.0 ± 2.1 ^a^	504.7 ± 7.8 ^a^	5.5 ± 0.03 ^ef^	6.5 ± 0.07 ^gh^	97.3 ± 0.4 ^ef^	0.41 ± 0.00 ^bcde^	1.95 ± 0.00 ^ab^
18	30 (−1)	80 (1)	5 (1)	27.5 ± 0.5 ^bc^	23.3 ± 1.0 ^b^	35.0 ± 0.6 ^b^	38.7 ± 0.3 ^a^	218.0 ± 9.5 ^bc^	5.6 ± 0.07 ^bcde^	6.7 ± 0.08 ^g^	96.6 ± 3.3 ^efg^	0.38 ± 0.00 ^ef^	1.94 ± 0.00 ^abc^
19	70 (1)	20 (−1)	1 (−1)	18.7 ± 1.2 ^de^	6.5 ± 0.8 ^d^	14.7 ± 0.2 ^i^	28.7 ± 0.2 ^efg^	95.9 ± 1.2 ^il^	5.8 ± 0.00 ^a^	7.7 ± 0.06 ^cd^	89.5 ± 0.5 ^il^	0.28 ± 0.01 ^h^	1.95 ± 0.01 ^ab^
20	50 (0)	50 (0)	3 (0)	16.3 ± 1.8 ^ef^	7.5 ± 0.1 ^cd^	22.4 ± 0.3 ^e^	33.2 ± 0.3 ^bc^	145.1 ± 2.9 ^e^	5.6 ± 0.04 ^cde^	7.8 ± 0.08 ^bcd^	92.7 ± 2.4 ^fghi^	0.38 ± 0.02 ^def^	1.95 ± 0.00 ^ab^
21	50 (0)	50 (0)	5 (1)	16.6 ± 0.5 ^ef^	9.5 ± 0.2 ^c^	24.4 ± 0.83 ^d^	33.6 ± 0.4 ^bc^	149.89 ± 17.2 ^e^	5.6 ± 0.03 ^abcd^	8.1 ± 0.10 ^a^	92.5 ± 0.7 ^ghi^	0.39 ± 0.01 ^cdef^	1.96 ± 0.00 ^a^

* Results are expressed as mean value ± standard deviation (n = 3). Different letters in the same column indicate significant differences resulting from ANOVA; *p* < 0.05—Tukey’s HSD post hoc test. DPPH^•^ (mg TE/g); ABTS^•+^ (mg TE/g); CUPRAC (mg TE/g); MCA (mg ETDAE/g); PMD (mmol TE/g); AChE (mg GALAE/g); BChE (mg GALAE/g); tyrosinase (mg KAE/g); α-amylase (mmol ACAE/g); α-glucosidase (mmol ACAE/g). Abbreviations: 2,2-difenil-1-picrylidrazyl (DPPH^•^); 2,2′-azino-bis-3-ethylbenzothiazoline-6-sulfonic acid (ABTS^•+^); cupric-reducing antioxidant capacity (CUPRAC); metal chelating activity (MCA); phosphomolybdenum (PMD); acetylcholine esterase (AChE); butyrylcholinesterase (BChE).

**Table 3:** Regression coefficients and statistical parameters that measure the correlation and significance of the models.

	Anthocyanins	Flavanols	Other Flavonoids	Flavonols	LMW Phenolics	Phenolic Acids	Stilbenes	Total Flavonoids	Total Phenolics	Carotenoids	Glucosinolates
	*y_1_*	*y_2_*	*y_3_*	*y_4_*	*y_5_*	*y_6_*	*y_7_*	*y_8_*	*y_9_*	*y_10_*	*y_11_*
β_0_	10.091 ***	40.721 ***	47.145 ***	64.091 ***	114.905 ***	197.446 ***	3.074 ***	162.049 ***	315.426 ***	233.914 ***	30.837 ***
β_1_	0.384	−0.141	4.681 •	4.015	2.923	−11.594	0.116	8.940	−8.553	−45.328 **	−1.108
β_11_	−0.059	0.097	2.165	−4.183 ***	0.359	−0.581	−0.069	6.386	−0.291	8.845	−0.690
β_2_	−1.339 **	−15.810 ***	27.989 ***	33.961	6.892	−48099 ***	−0.873 ***	44.801 ***	−42.880 **	113.231 ***	0.148
β_22_	−2.012 **	−18.742 ***	15.950 **	18.093 **	−0.584	−76.935 ***	−0.154	13.288	−77.674 ***	−59.995 *	−14.386 ***
β_3_	0.583	0.175	5.016	1.658	2.698	−8.892	0.019	7.434	−6.174	−33.469 •	−1.578 •
β_33_	0.710	0.268	7.360 •	9.841	−4.310	4.865	−0.006	18.180 •	0.548	−2.789	−0.083
β_12_	0.247	2.352	11.421	14.756 *	4.588	16.391	−0.039	28.778 *	20.939	−17.312	2.572 •
β_13_	−0.115	1.327	−4.227	−5.299	−0.419	10.429	−0.287	−8.315	9.723	40.734	1.261
β_23_	0.156	3.874	8.270 •	9.127	11.419	−4.696	0.113	21.428 •	6.835	6.095	2.891 *
R^2^	0.7086	0.8561	0.9015	0.8587	0.1836	0.8548	0.8352	0.7973	0.7545	0.8471	0.9194
*p*-value	0.0053	0.0000	0.0000	0.0000	0.9173	0.0000	0.0000	0.0004	0.0016	0.0000	0.0000

Significance codes: 0.000 ***; 0.001 **; 0.01 *; 0.05 •.

**Table 4:** Regression coefficients and statistical parameters that measure the correlation and significance of the models.

	DPPH^•^	ABTS^•+^	CUPRAC	MCA	PMD	AChE	BChE	Tyrosinase	α-Amylase	α-Glucosidase
	*y_12_*	*y_13_*	*y_14_*	*y_15_*	*y_16_*	*y_17_*	*y_18_*	*y_19_*	*Y_20_*	*y_21_*
β_0_	15.986 ***	7.187 ***	21.832 ***	31.499 ***	138.590 ***	5.636 **	7.807 **	96.065 ***	0.401 ***	1.947 ***
β_1_	1.102 **	2.439 ***	1.522 **	0.703	20.688	−0.007	−0.201 **	0.147	0.001	−0.016 •
β_11_	0.593 •	0.730	−0.286	0.178	8.503	0.003	−0.092	−1.325	−0.009	−0.016 *
β_2_	5.761 ***	9.767 ***	9.348 ***	4.081 ***	64.141 ***	−0.058 *	−0.597 ***	3.679 •	0.047 ***	−0.017
β_22_	5.319 ***	4.479 ***	2.650 **	1.115	21.499	−0.027	−0.370 **	1.680	−0.036 *	−0.008
β_3_	−0.380	−0.772	−0.364	0.943	−16.171	−0.005	−0.100	2.720	−0.009	−0.002
β_33_	−0.270	1.061	0.847	1.277	14.691	0.009	0.085	1.570	0.001	−0.001
β_12_	−1.178 •	0.095	0.875	2.164 *	38.159 •	−0.016	−0.195 •	5.287 •	0.035 *	−0.001
β_13_	−0.764	0.308	0.039	0.140	−33.900	−0.013	−0.008	0.534	0.010	−0.019
β_23_	−0.658	1.824	1.220	1.449	−17.649	−0.022	−0.233 •	1.566	0.0334 *	−0.008
R^2^	0.9572	0.9376	0.9564	0.8063	0.7359	0.5343	0.857	0.4677	0.7805	0.4023
*p*-value	0.0000	0.0000	0.0000	0.0003	0.0027	0.1024	0.0000	0.2090	0.0007	0.3607

Significance codes: 0.000 ***; 0.001 **; 0.01 *; 0.05 •.

**Table 5:** Most discriminant compounds highlighted by VIP selection method following the OPLS-DA modelling and as a function of the % of ethanol (discriminant factor).

Discriminant Compounds	Chemical Class	VIP Score	Marker
2′-apo-beta-carotenal	Carotenoids	1.68 ± 0.10	80% ethanol
Methyl 3-(methylthio)butanoate	Glucosinolates	1.82 ± 0.10	50% ethanol
7-hydroxysecoisolariciresinol	Phenolic compounds	1.73 ± 0.19	50% ethanol
1′-carboxy-gamma-tocotrienol	Tocotrienols	1.72 ± 0.33	50% ethanol

**Table 6:** Canonical analysis used to predict the maximum response values among all the predictor combinations at d = ±5.

Dependent Variables		Temperature (°C)	Ethanol (%)	Ultrasound Power (W)	Estimated Yield/Activity	
*y_1_*	Anthocyanins	43.92	43.34	294.48	28.15	μg/g
*y_2_*	Flavanols	25.08	30.95	0.48	51.85	μg/g
*y_3_*	Other flavonoids	58.98	97.91	98.88	154.68	μg/g
*y_4_*	Flavonols	60.30	97.52	101.04	190.25	μg/g
*y_6_*	Phenolic acids	98.06	44.63	251.28	296.77	μg/g
*y_7_*	Stilbenes	−3.98	−0.70	238.56	5.12	μg/g
*y_8_*	Total flavonoids	39.56	96.14	102.48	272.23	μg/g
*y_9_*	Total phenolics	22.16	35.24	6.00	376.14	μg/g
*y_10_*	Total carotenoids	2.60	83.69	12.00	638.08	μg/g
*y_11_*	Total glucosinolates	33.52	44.69	12.48	34.94	μg/g
*y_12_*	DPPH^•^	24.64	90.89	90.96	35.15	mg TE/g
*y_13_*	ABTS^•+^	11.72	93.56	198.48	39.31	mg TE/g
*y_14_*	CUPRAC	50.86	86.93	202.80	46.88	mg TE/g
*y_15_*	MCA	32.24	92.63	113.52	39.78	mg ETDAE/g
*y_16_*	PMD	70.38	95.06	31.68	446.88	mmol TE/g
*y_17_*	AChE	2.78	34.88	106.32	5.71	mg GALAE/g
*y_18_*	BChE	49.14	1.61	244.08	9.89	mg GALAE/g
*y_19_*	Tyrosinase	60.54	93.83	73.44	108.98	mg KAE/g
*y_20_*	α-Amylase	82.90	98.03	136.50	0.54	mmol ACAE/g
*y_21_*	α-Glucosidase	16.86	1.10	219.60	2.07	mmol ACAE/g

Abbreviations: 2,2-difenil-1-picrylidrazyl (DPPH^•^); 2,2′-azino-bis-3-ethylbenzothiazoline-6-sulfonic acid (ABTS^•+^); cupric-reducing antioxidant capacity (CUPRAC); metal chelating activity (MCA); phosphomolybdenum (PMD); acetylcholine esterase (AChE); butyrylcholinesterase (BChE); LMW: low-molecular-weight phenolics. Note: y_5_ low-molecular-weight phenolics were not considered in this analysis because they were not significant in the RSM response (*p* > 0.05).

## Data Availability

The data presented in this study are available in the article and Supplementary Materials.

## Supplementary Materials

The following supporting information can be downloaded at: https://www.mdpi.com/article/10.3390/antiox12020313/s1, Figure S1: Three-dimensional response surface plots carried out considering the (A) anthocyanins, (B) flavanols, (C) other flavonoids, (D) flavonols, (E) low-molecular-weight (LMW) phenolics, (F) phenolic acids, (G) stilbenes, (H) total flavonoids, (I) total phenolics, (J) total carotenoids, (K) total glucosinolates phytochemical profile of the 21 duckweed extracts, as a function of extraction temperature, ultrasound power, and % of ethanol. Figure S2: Three-dimensional response surface plots carried out considering the (A) DPPH^•^, (B) ABTS^•+^, (C) CUPRAC, (D) MCA, (E) PMD, (F) AChE, (G) BChE, (H) tyrosinase, (I)), α-amylase, and (J) α-glucosidase antioxidant activities and enzyme inhibition capacities of the 21 duckweed extracts, as a function of extraction temperature, ultrasound power, and % of ethanol. Figure S3: Two-dimensional contour plots carried out considering the (A) anthocyanins, (B) flavanols, (C) other flavonoids, (D) flavonols, (E) low-molecular-weight (LMW) phenolics, (F) phenolic acids, (G) stilbenes, (H) total flavonoids, (I) total phenolics, (J) total carotenoids, (K) total glucosinolates phytochemical profile of the 21 duckweed extracts, as a function of extraction temperature, ultrasound power, and % of ethanol. Figure S4: Two-dimensional contour plots carried out considering the (A) DPPH^•^, (B) ABTS^•+^, (C) CUPRAC, (D) MCA, (E) PMD, (F) AChE, (G) BChE, (H) tyrosinase, (I)), α-amylase, and (J) α-glucosidase antioxidant activities and enzyme inhibition capacities of the 21 duckweed extracts, as a function of extraction temperature, ultrasound power, and % of ethanol. Table S1: excel file containing the following information: (a) the sample legend, (b) dataset of compounds annotated by UHPLC-HRMS, (c) annotation information, (d) MSMS confirmation in QC samples, (e) OPLS-DA validation parameters and VIP marker compounds as a function of % of ethanol. Table S2: Pearson’s correlation coefficients between the phytochemical composition of duckweed extracts and their bioactive properties.
