# Supplementary material for: The Combination of Untargeted Metabolomics with Response Surface Methodology to Optimize the Functional Potential of Common Duckweed (Lemna minor L.)
Source: Antioxidants (Basel). 2023 Jan 29;12(2):313. doi: 10.3390/antiox12020313 (PMC9952736; doi:10.3390/antiox12020313)
Supplement: Supplementary file 1 [file antioxidants-12-00313-s001.zip › Supplementary Figures.pdf]

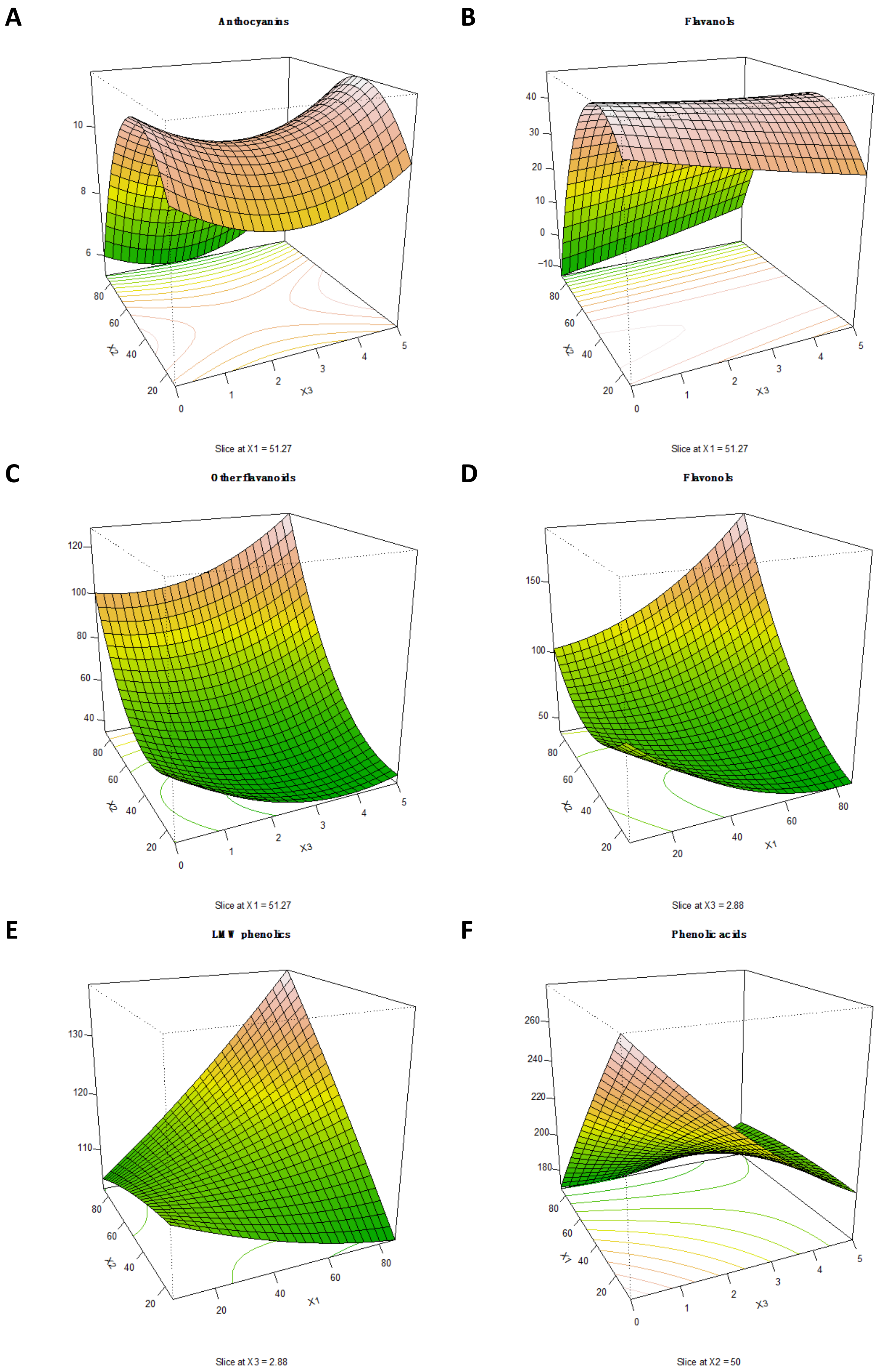
A
Anthocyanins
Slice at X1 = 51.27
B
Flavanols
Slice at X1 = 51.27
C
Other flavanoids
Slice at X1 = 51.27
D
Flavonols
Slice at X3 = 2.88
E
LMW phenolics
Slice at X3 = 2.88
F
Phenolic acids
Slice at X2 = 50

G

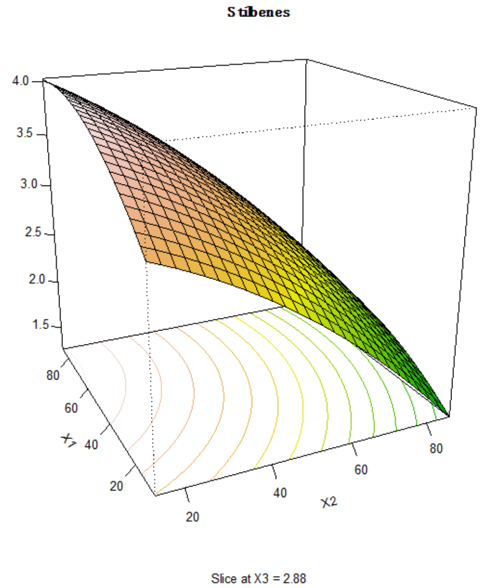
Stilbenes
4.0
3.5
3.0
2.5
2.0
1.5
X1
20
40
60
80
X2
20
40
60
80
Slice at X3 = 2.88

H

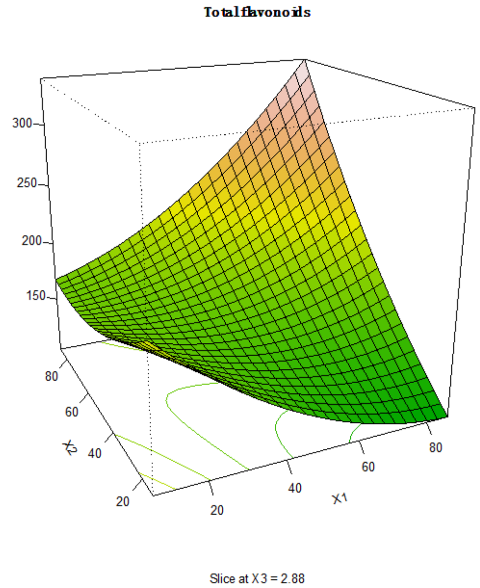
Total flavonoids
300
250
200
150
X2
20
40
60
80
X1
20
40
60
80
Slice at X3 = 2.88

I

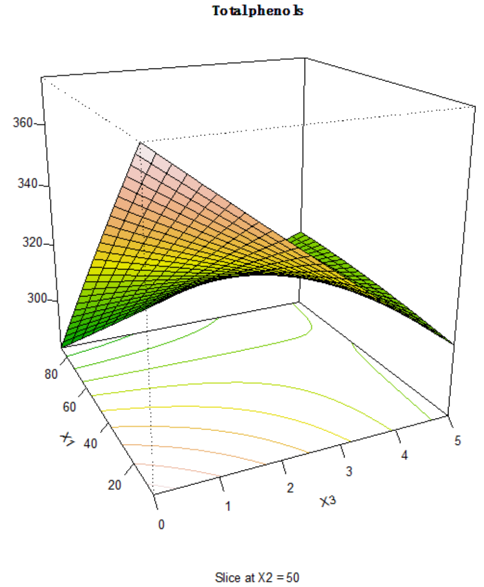
Total phenols
360
340
320
300
X1
20
40
60
80
X3
0
1
2
3
4
5
Slice at X2 = 50

J

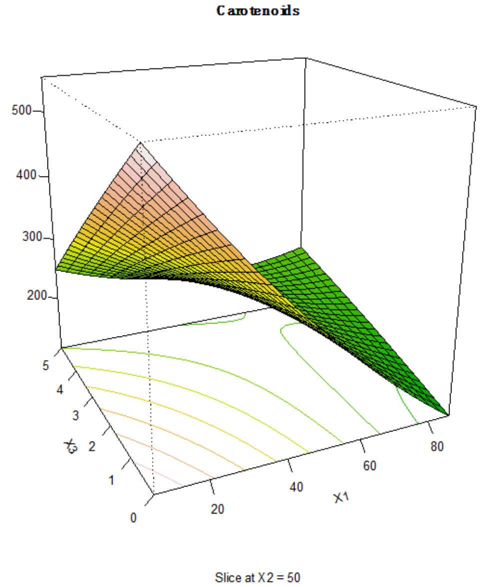
Carotenoids
500
400
300
200
X3
0
1
2
3
4
5
X1
20
40
60
80
Slice at X2 = 50

K

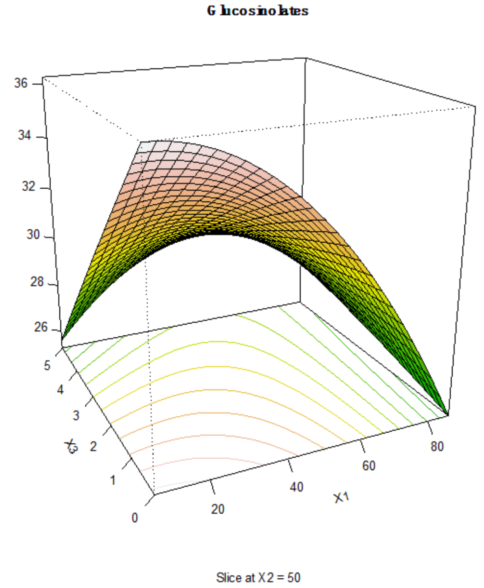
Glucosinolates
36
34
32
30
28
26
X3
0
1
2
3
4
5
X1
20
40
60
80
Slice at X2 = 50

Figure S1. Three-dimensional response surface plots carried out considering the (A) Anthocyanins, (B) flavanols, (C) other flavonoids, (D) flavonols, (E) Low-molecular-weight (LMW) phenolics, (F) phenolic acids, (G) stilbenes, (H) total flavonoids, (I) total phenolics, (J) total carotenoids, (K) total glucosinolates phytochemical profile of the 21 duckweed extracts, as a function of extraction temperature, ultrasound power, and % Ethanol.

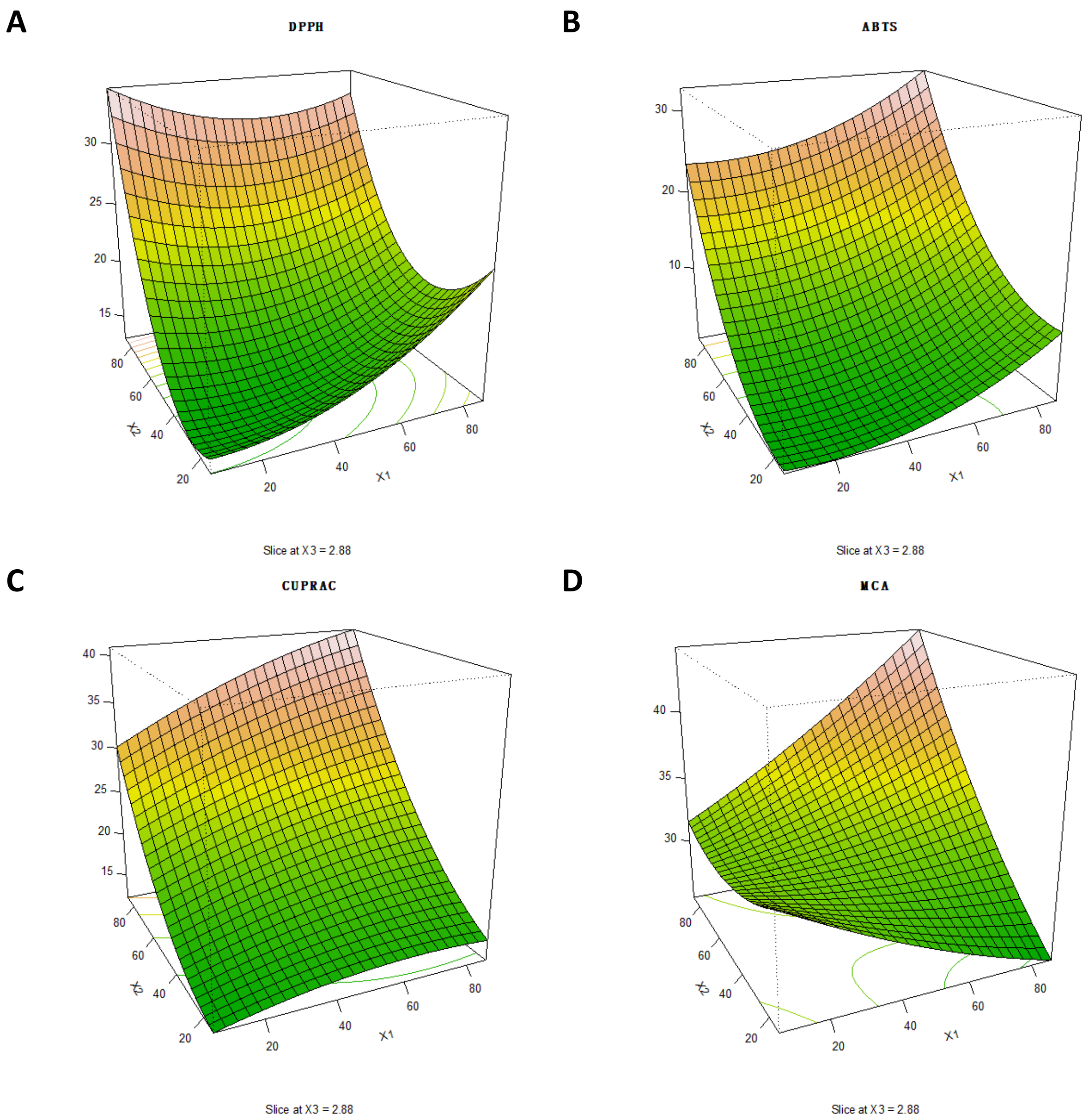

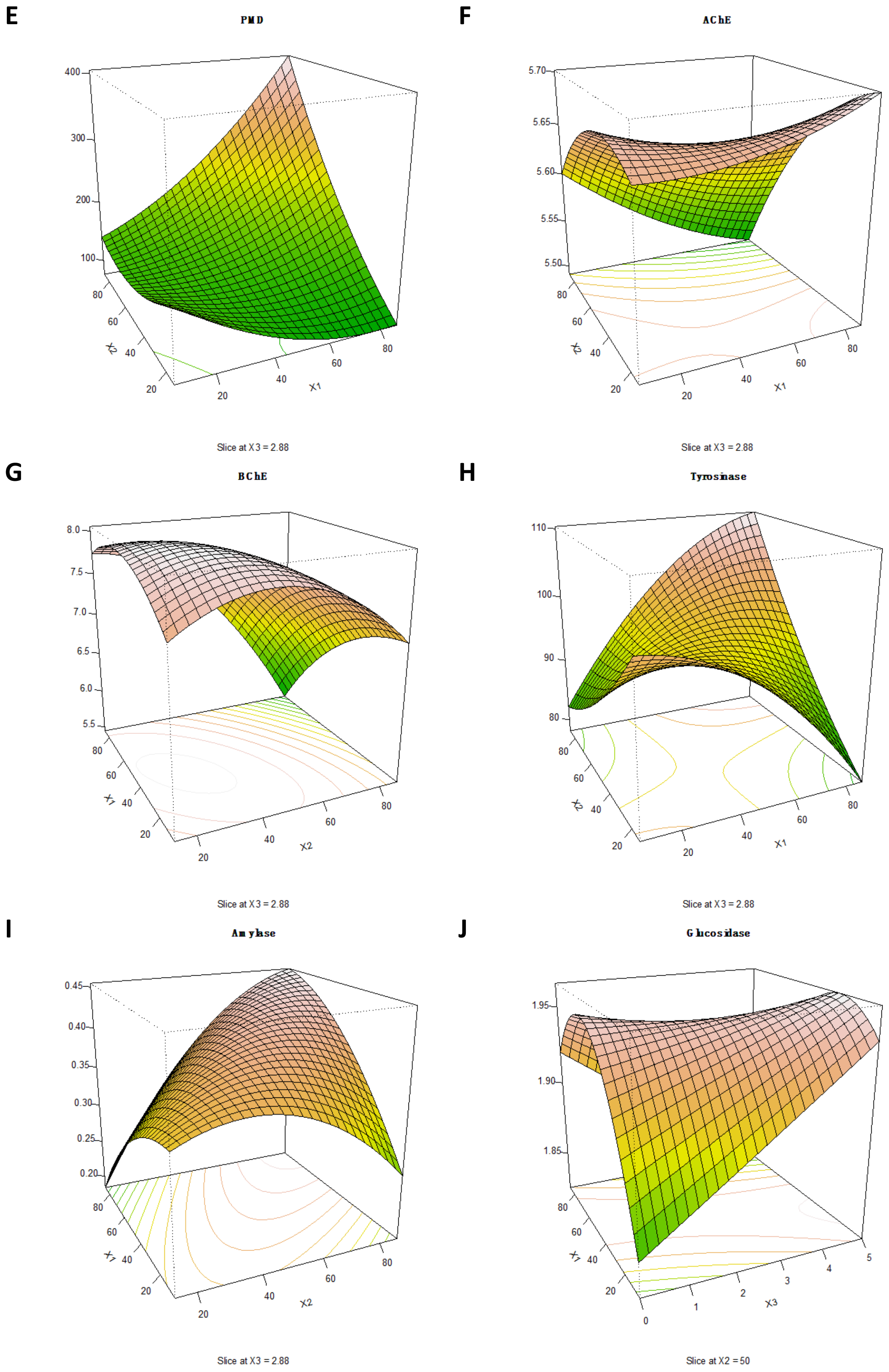
E
PMD
400
300
200
100
80
60
X2
40
20
20
40
60
80
X1
Slice at X3 = 2.88
F
AChE
5.70
5.65
5.60
5.55
5.50
80
60
X2
40
20
20
40
60
80
X1
Slice at X3 = 2.88
G
BChE
8.0
7.5
7.0
6.5
6.0
5.5
80
60
X1
40
20
20
40
60
80
X2
Slice at X3 = 2.88
H
Tyrosinase
110
100
90
80
80
60
X2
40
20
20
40
60
80
X1
Slice at X3 = 2.88
I
Amylase
0.45
0.40
0.35
0.30
0.25
0.20
80
60
X1
40
20
20
40
60
80
X2
Slice at X3 = 2.88
J
Glucosidase
1.95
1.90
1.85
80
60
X1
40
20
0
1
2
3
4
5
X3
Slice at X2 = 50

Figure S2. Three-dimensional response surface plots carried out considering the (A) DPPH, (B) ABTS, (C) CUPRAC, (D) MCA, (E) PMD, (F) AChE, (G) BChE, (H) Tyrosinase, (I)), alpha-Amylase, and (J) alpha-Glucosidase, antioxidant activities and enzyme inhibition capacities of the 21 duckweed extracts, as a function of extraction temperature, ultrasound power, and % Ethanol.

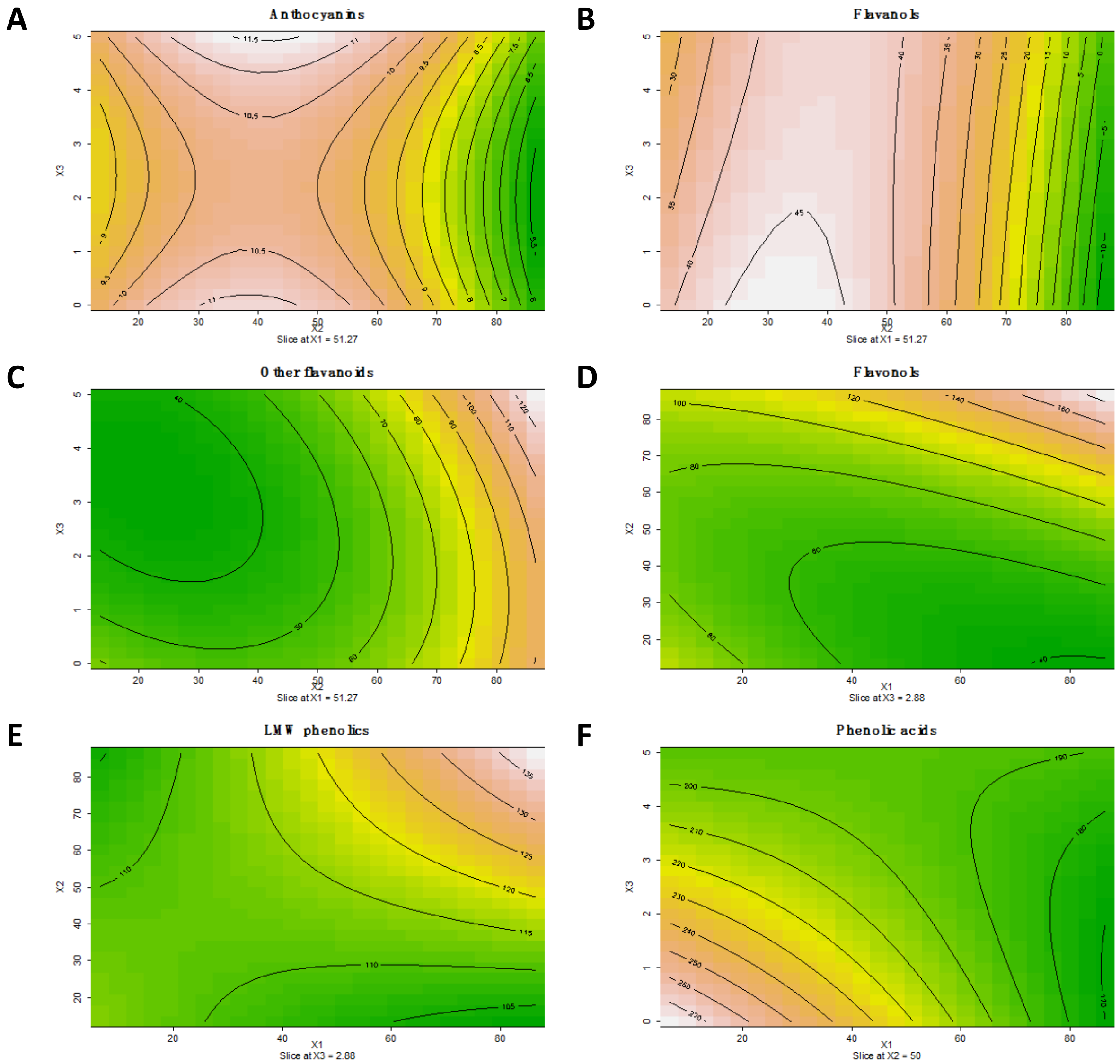

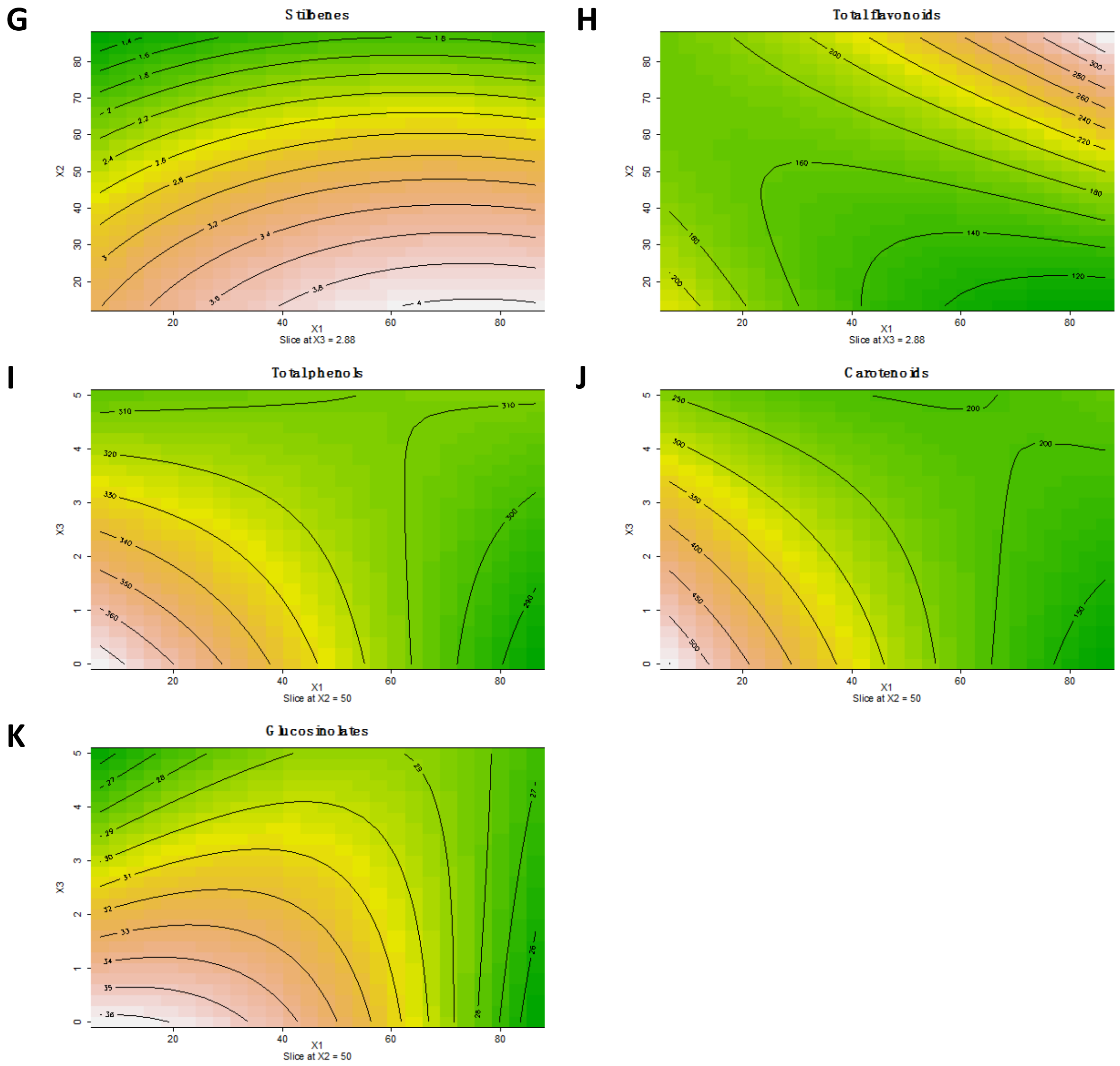

Figure S3. Two-dimensional contour plots carried out considering the (A) Anthocyanins, (B) flavanols, (C) other flavonoids, (D) flavonols, (E) Low-molecular-weight (LMW) phenolics, (F) phenolic acids, (G) stilbenes, (H) total flavonoids, (I) total phenolics, (J) total carotenoids, (K) total glucosinolates phytochemical profile of the 21 duckweed extracts, as a function of extraction temperature, ultrasound power, and % Ethanol.

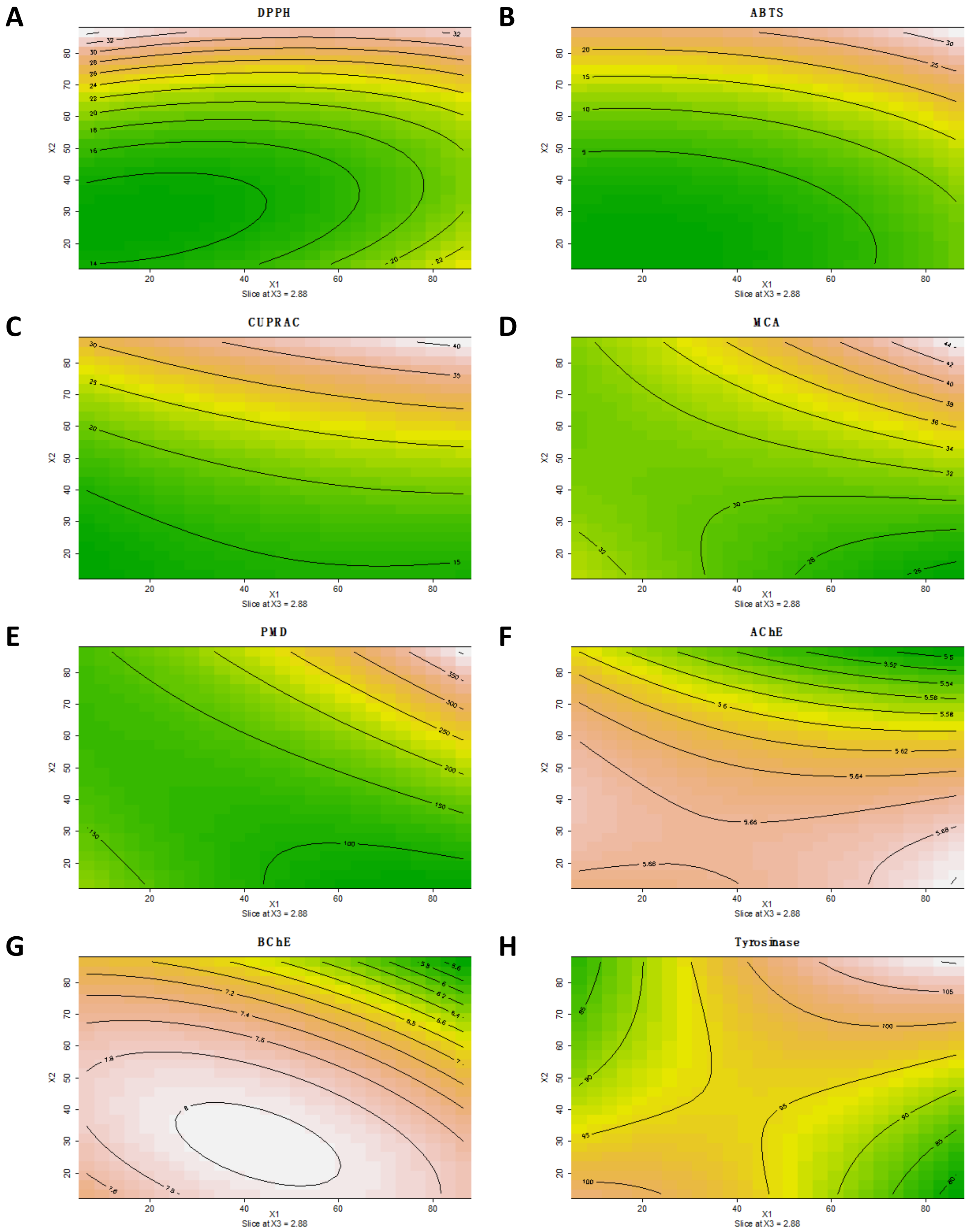
A
DPPH
B
ABTS
C
CUPRAC
D
MCA
E
PMD
F
AChE
G
BChE
H
Tyrosinase
X1
X2
Slice at X3 = 2.88

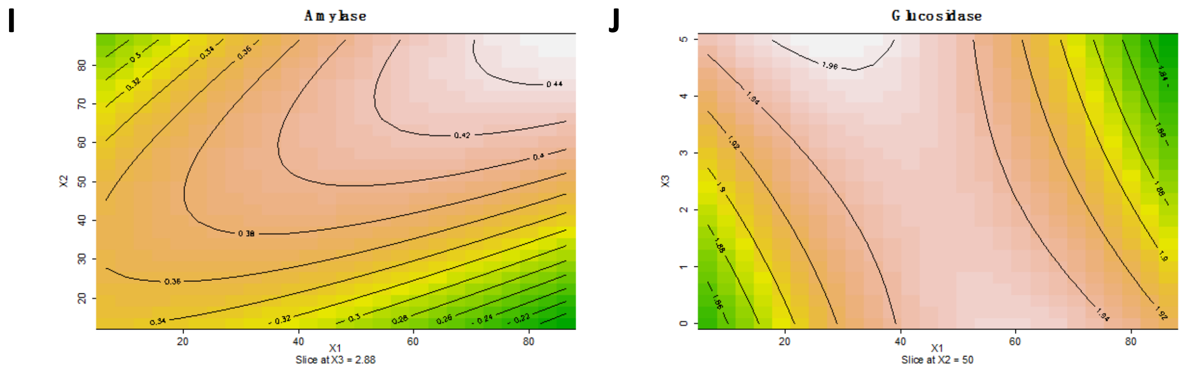

Figure S4. Two-dimensional contour plots carried out considering the (A) DPPH, (B) ABTS, (C) CUPRAC, (D) MCA, (E) PMD, (F) AChE, (G) BChE, (H) Tyrosinase, (I)), alpha-Amylase, and (J) alpha-Glucosidase, antioxidant activities and enzyme inhibition capacities of the 21 duckweed extracts, as a function of extraction temperature, ultrasound power, and % Ethanol.
